# Distributed uplink cache for improved energy and spectral efficiency in B5G small cell network

**DOI:** 10.1371/journal.pone.0268294

**Published:** 2022-05-17

**Authors:** Mubarak Mohammed Al Ezzi Sufyan, Waheed Ur Rehman, Tabinda Salam, Abdul Rahman Al-Salehi, Qazi Ejaz Ali, Abdul Haseeb Malik

**Affiliations:** 1 Department of Computer Science, University of Peshawar, Peshawar, Pakistan; 2 Department of Computer Science, Shaheed Benazir Bhutto Women University Peshawar, Peshawar, Pakistan; 3 Department of Electronics Engineering, International Islamic University, Islamabad, Pakistan; RMIT University, AUSTRALIA

## Abstract

The advent of content-centric networks and Small Cell Networks (SCN) has resulted in the exponential growth of data for both uplink and downlink transmission. Data caching is considered one of the popular solutions to cater to the resultant challenges of network congestion and bottleneck of backhaul links in B5G networks. Caching for uplink transmission in distributed B5G scenarios has several challenges such as duplicate matching of contents, mobile station’s unawareness about the cached contents, and the storage of large content size. This paper proposes a cache framework for uplink transmission in distributed B5G SCNs. Our proposed framework generates comprehensive lists of cache contents from all the Small Base Stations (SBSs) in the network to remove similar contents and assist uplink transmission. In addition, our framework also proposes content matching at a Mobile Station (MS) in contrast to an SBS, which effectively improves the energy and spectrum efficiency. Furthermore, large size contents are segmented and their fractions are stored in the distributed cache to improve the cache hit ratio. Our analysis shows that the proposed framework outperforms the existing schemes by improving the energy and spectrum efficiency of both access and core networks. Compared to the existing state of the art, our proposed framework improves the energy and spectrum efficiency of the access network by 41.28% and 15.58%, respectively. Furthermore, the cache hit ratio and throughput are improved by 9% and 40.00%, respectively.

## 1 Introduction

In recent years, content-based services such as video streaming are exponentially growing. In addition, the number of internet users by 2023 is expected to be 5.3 billion [1]. This will tremendously increase the network volume for both uplink and downlink in Beyond 5th Generation (B5G) networks. It will also result in several challenges such as traffic load, congestion, and latency, in addition to, significant consumption of energy and spectrum [2–4]. To solve the challenges incurred by this data explosion, the SCN is widely advocated in B5G networks. However, it affects the users’ data requirements over time and location. In addition to, creating bottlenecks at the backhaul. It is caused as a result of a significantly improved data transmission rate in the SCN. Another way to cope with the data explosion is the use of caching in the network. In this technique, cached versions of the contents are stored in the network at different locations such as Base Station (BS), Macro Base Station (MBS), SCN, and/or Mobile Stations (MSs). If the cached versions are available, devices do not need to upload or download the contents to/from the core network. In addition, as the contents are available locally, it reduces the cost of communication, energy/bandwidth consumption and latency [5–8].

The rapid growth of the SCNs also increases the complexity of the cellular network, especially in a cooperative scenario. In such a case, the distributed cache can be employed to act as a single cache but span across the network. A distributed cache can be seen as an extension of a single cache. It provides a higher data rate with less energy and bandwidth consumption along with low latency to access contents in order to meet users’ quality of experience [9–11]. Distributed caching may be employed for both downlink and uplink transmission depending upon the demands of the MSs [12]. Downlink caching restrains the unnecessary download of content by providing it locally based on its popularity to reduce the congestion on the backhaul link and core network. Whereas, uplink caching is used to store the MS’s data intended to be uploaded with the constraints of avoiding unnecessary upload to reduce the data traffic in the network, especially the access network link [13, 14]. Distributed caching can be deployed in different types of network such as content-centric network [15], cloud-small cell networks [16, 17], wireless cellular networks [18], dense-SBSs [19], an edge network [20], B5G mobile edge computing [6], HetNets SBs [21], B5G relaying networks [22], Small Cell Base Station (SCBS) [23], multi-antenna SCNs [24], and 5G-SBS [25].

Recently, a lot of research work is done on addressing the challenges of distributed caching in a cellular network with some work on uplink caching [26–33]. The authors in [26], proposed a novel upload cache architecture to support the parallel uploading of segmented files. The authors in [27], studied the energy-efficient cooperative coded caching issue in heterogeneous SCNs to minimize the energy consumption for content caching and content delivery. The authors in [28], proposed an uplink cache system in delay tolerant SCNs. The main focus in [28] is to analyze the cache size and its effectiveness for uplink caching. Furthermore, duplicate elimination of contents is performed at SBS via matching the hash key of chunks of the file after uploading the real content, which is not very practical for an uplink scenario. The authors in [29] developed a novel multiple-input multiple-output (MIMO) network architecture with a large number of BSs employing cache-enabled uplink transmission. The authors proposed the modified von Mises distribution as a popularity distribution function and derive the outage probability, in addition, identified the relationship between cache storage and outage probability to be directly proportional. Therefore, it is found that the delivery rate also improves with the increase of cache storage space in addition to a denser network. The authors in [30] have proposed an innovative approach to reduce peak traffic by utilizing distributed cache of Internet of Things (IoT) devices. The proposed scheme employed an uplink transmission scheduling based on delay adaptation to mitigate the sporadic access network congestion. The average delivery latency is investigated by the authors in [31], where they proposed a proactive caching technique for movie on-demand streaming on the internet. The probabilistic caching is studied in [32], where authors proposed an optimized caching strategy to improve the successful download probability. Furthermore, in [33], the authors presented the contents matching the mobile data to be uploaded at an MS level before the real transmission of the actual content taken place to uploading the dissimilar contents only without considering the cooperative scenario.

All the aforementioned research papers proposed novel uplink cache architectures and schemes to effectively improve the network performance. However, these papers have not presented the cache benefits on Energy Efficiency (EE) and Spectral Efficiency (SE). In addition, the existing literature proposes to use an SBS for performing content matching [28, 29], which entails that the MSs will be unaware of cached contents. This will lead to unnecessary upload of content, which is not desirable. Furthermore, the existing literature has also identified that the large content size significantly degrades the performance of the distributed cache. In addition, the existing works in [28, 29] have not considered content duplication and its effect on energy and spectral efficiency in a distributed scenario. The authors in [33] considered content matching at an MS mitigating the duplicate upload, however, it did not consider a distributed scenario. As well as, the content segmentation and its effective placement in the context of distributed cache is not considered in the existing literature. This motivates us to propose a novel cache-enabled uplink transmission framework that addresses all these challenges. The main objective of this work is to improve energy and spectral efficiency along with improving cache performance in a distributed scenario. Firstly, our proposed framework generates an unduplicated list of cache contents in the distributed scenario in an attempt to be used as a map for an MS to decide whether to upload the contents or not. Secondly, it proposes the scheme to perform content matching at an MS and lastly, it performs the content segmentation to divide a large size content into smaller for effective storage across the distributed network. The main contributions of this paper can be summarized as follows:

1. We proposed a novel scheme to effectively generate a disparate list of distributed cache for uplink transmission. The proposed scheme leverages cooperative communication to efficiently generate a list of contents based on popularity and content validity. This way the list is updated with the most relevant content, which improves the cache hit ratio.
2. An efficient content matching scheme is also proposed to facilitate an MS to corroborate the cached contents. This will significantly limit the amount of uplink transmission, which will improve the EE and SE.
3. We also proposed a content segmentation with distributed placement scheme, which improves the content placements in the distributed cache network by splitting the larger contents into smaller. The proposed scheme significantly improves the cache hit ratio, which intrinsically improves the EE and SE.
4. We compare our proposed framework with state-of-the-art architectures and algorithms to analyze its effectiveness. We mainly focused on energy consumption and spectrum efficiency, which is the main limiting factor of the existing literature.

This paper is organized as follows: Related work is discussed in Section 3. The system model is discussed in Section 4. Section 5 presented our proposed framework. Experimental, Performance evaluation and conclusion are discussed in Sections 6 and 7, respectively.

## 2 Related work

The use of cache for the downlink transmission is widely researched. Its effectiveness is broadly accepted, especially the energy and spectral efficiencies, which is summarized in Table 1, with some work related to distributed cache in Table 2. However, cache-enabled uplink is researched to a lesser extent. The state-of-the-art and our reference points are in [28, 29]. In [28], the authors presented a framework to relieve the burden of wireless SCNs by considering cache-enabled uplink transmission in a delay-tolerant network. The paper also proposed duplicate content matching at an SBS by comparing the hash key of chunks of the file after uploading real content. The authors also used First-Input-First-Output (FIFO), random and probabilistic content scheduling strategies for cache management. It enabled an SBS to eliminate the redundancy among users’ uploaded contents to improve the network transmission efficiency. Similarly, in [29], the authors presented a cache-uplink framework for HetNet with stochastic distributed BSs for temporary caching of user-generated contents. The authors also presented the relationship between cache storage space and outage probability. While the authors in [33], presented a Broadcast cache assist uplink (BCAU) scheme to perform a matching among the attributes of new content and cache contents at an MS to discard the uploading of the available content in the SBS cache before the real transmission of the actual content taken place to improve the EE and SE of uplink transmission over B5G-SCN but the authors is not considering the cooperative among the distributed cache. However, these papers have not considered EE and SE of distributed cache. In addition, content matching at an SBS, as proposed in [28, 29, 33], entails unnecessary uploads from the MSs. Lastly, the challenges of large size contents are not addressed in these papers.

**Table 1:** Summary of existing works about the impact of cache on energy, spectral and cache efficiencies of SCN.

Ref.	Year	Caching at	Contributions	Components	Modeling Tools	Performance Measurements
**Energy Efficiency**						
[27]	2017	SBS, MBS	Proposed a cooperative coded caching scheme to obtain EE by reducing the Energy Consumption (EC) by content caching and delivery, and transport of cooperative, and backhaul, in addition, proposed a greedy-based caching placement algorithm to optimize the caching placement that improved the content delivery efficiency and enhanced the Quality of experience (QoE) for end-users.	Het.SCN consists of SBS, MBS, Users	Maximum-Distance Separable (MDS), Zipf	EC
[34]	2017	SCBS	Investigated the system throughput by analyzing the EE of cache-enabled cooperative Dense-SCNs (D-SCNs) using an affinity propagation algorithm to divide the SCBSs into different clusters and derivation the closed-form of the EE of Coordinated Multi-Point-Joint Transmission (CoMP-JT) cache-enable cooperative D-SCNs to reduce the EC of transmitting, circuit powers, and caching.	D-SCN consists of SCBS, MBS, User	PPP	EE
[35]	2018	MBS, SBS	Proposed a Scalable Video Coding (SVC) scheme based fractional caching for providing on-demand High Definition Video (HDV) with different perceptual qualities to solve the issue of repeated content deliveries and then derivation the expressions for successful transmission probabilities and ergodic service rates based on stochastic geometry to establish the integrated power consumption model and using the standard gradient projection method to optimize EE.	HetNets consist of MBS, SBS, Users	Stochastic Geometry, hPPP, Zipf	Successful transmission probability, EE
[36]	2019	MBS, SBS	Proposed an energy-efficient collaborative caching scheme to minimize the energy consumption by considering the constraints of limited storage capacity, content popularity, placement, and access to contents to provide efficient utilization of hotspot cache, and using knapsack with Zipf to ensure the local availability of cache contents and minimizing transportation cost. In addition, the proposed scheme is to support the Device-to-Device(D2D) communication.	5G-WN consist of MBS, SBS, Users	Zipf and Knapsack	EC of backhaul, hotspot, and the overall network, Transport energy cost
[37]	2019	MBS, SBS	Investigated a file placement strategy in cooperative SCN to maximized EE via proposed an energy-efficient cooperative caching scheme (EECCS) with the capability of accessing the SBS cache contents by other SBSs to ensure the caching service for all the users.	SCN consists of MBS, SBS, users	Additive White Gaussian Noise (AWGN)	EE
[38]	2020	MBS, SBS	Propose a sleeping mechanism in the 5G-SCNs with Energy Harvesting (EH) function with jointly cooperative caching to minimize EC and backhaul traffic by the binary particle swarm optimization (BPSO) to turn the SBS to sleep mode at every time slot based on the battery capacity and fewer contents delivery, in addition, an Iterative Algorithm With Status Update and Cooperative Caching to optimize the content caching placement.	5G-SCN consist of MBS, SBS, Users	Zipf, MDS	EC of SBSs, backhaul and power grid
[39]	2020	MBS, mmSBS, and UE	Analyzed the successful probability for users to obtain files by different communication modes and consider the energy costs and offloading probability as the objective function to optimize the joint caching policy (Random caching strategy (RCS), and File popularity-based random strategy (FPRS)) of the user equipment (UE) and mmWave SBSs (mmSBSs) to improve the file transmission efficiency.	MBS, mmSBS, UE	RCS, FPRS, Zipf, hPPP	Energy cost and Offloading Probability
**Spectral Efficiency**						
[40]	2017	Helper, Pico-BS	Investigated the optimal caching policy to maximize Area Spectral Efficiency (ASE) and success probability of cache-enabled HetNets under the framework of the probability caching to obtain the optimal caching probability and then analyzed the impact of the parameters of the critical system to compare the ASE with the traditional HetNets.	HetNet consist of Pico BSs, MBS, Helper	Stochastic Geometry, hPPP, Zipf	Success Probability, Cache Probability, ASE
[41]	2018	SCBS	Presented an analytical framework to evaluate the SE of content caching and retrieving in Ultra-D-SCN via proposing the probabilistic content retrieving (PCR) strategy to improve the probability of serving the user, in addition to studied the optimization of the content retrieving probability (CRP) to avoid the degradation of the ASE even to be zero with the density of SCNs and users.	U-D-SCN consist of SCBS, users	hPPP	ASE
[42]	2018	SBS	Studied the transmission mode selection to mitigate the inter-cell interference by CoMP-JT to allow multiple SBSs to transmit simultaneously and the caching scheme to minimize wireless backhaul resources consumption by optimizing the caching placement while guaranteeing the SE requirement.	SCN consist of SBS, UE	Zipf, PBCS, RCS	Backhaul resource consumption
[43]	2020	SBS	Studied the cache placement problem and Cooperative Multi-Point (CoMP) as CoMP-aware caching with the SE guaranteed. For that, they proposed corresponding algorithms, a Boundary Function to balance the traffic load for solving the transmission mode selection, a distributed backtracking algorithm with low complexity to save more backhaul resources consumption by finding a sub-optimal caching solution for the SBSs caching, and Optimize the radio resource allocation of access and wireless backhaul links via a resources allocation algorithm to minimize the average downloading delay.	SCN consists of SBS, UE	Backtracking method	Download delay, Backhaul traffic load
[44]	2020	MBS, SBS	Proposed the Backhaul spectrum occupation that contains all files and connects to the mobile core network to provide wireless backhaul connections to the SBSs via broad mmWave spectrum to improve the network throughput in the existing HetNets.	MBSs, SBSs, Mobile	PPPs, Zipf	Average Potential Throughput, Spectrum Transfer
**Cache efficiency**						
[45]	2020	SBS	Presented the optimizing cooperative content caching and recommendation in SCNs to maximize the gain of the cache by focusing on an online and distributed content replacement to content caching since SBS content caching kept pace with the dynamic content recommendation.	SBS, User	Continuous Time Markov chain (CTMC), LRU, FIFO, Zipf	Cache hit ratio
[46]	2021	SBS	Propose the Mobile Edge Computing (MEC) assisted flexible transcoding strategy to achieve adaptive bitrate video streaming to provide viewers with low latency by caching the proper bitrate version of the video segments at the edge servers and selecting the appropriate bitrate version of the video segments to perform transcoding under joint considering access control, resource allocation, and user preferences.	SBS, UE	NOMA, AWGN	Delay, Cache hit ratio

**Table 2:** Summary of cooperative distributed cache methods.

Ref.	Cache placed	Proposed Scheme
[16]	SCN	Maximum Distance Separable (MDS) Code.
[47]	5G-SCBS	Decentralized Collaborative Video Caching.
[48]	SBS	Encoded into multiple resolutions with multiple Bitrate videos.
[49]	Wireless net	Rolling-horizon collaborative cache optimization scheme.
[50]	MSNs	Social-aware cooperative caching mechanism.
[51]	MNC	A reinforcement learning (RL)-based online learning algorithm to search the optimal caching policy.
[52]	MEC	A Group Behavior and Popularity Prediction based Collaborative Caching (GPCC) strategy based on UDR (User Detail Record).
[48]	SBS	Cooperative caching at the edge in SBS based on a Bayes-based learning algorithm.
[53]	ICN	Edge-oriented Collaborative Caching (ECC).
[54]	MEN	Hybrid collaborative caching (hy-CoCa).
[55]	SCBS	A rolling horizon cache optimization scheme.
[56]	SBSs	Collaborative content caching among multiple SBSs.
[57]	HetsNet	Spatially cooperative caching strategy for a two-tier HetsNet consisting of edge servers and caching helpers.
[58]	SBS	A backhaul-based cooperative caching scheme that groups several SBSs.

## 3 System model

**Fig 1:**
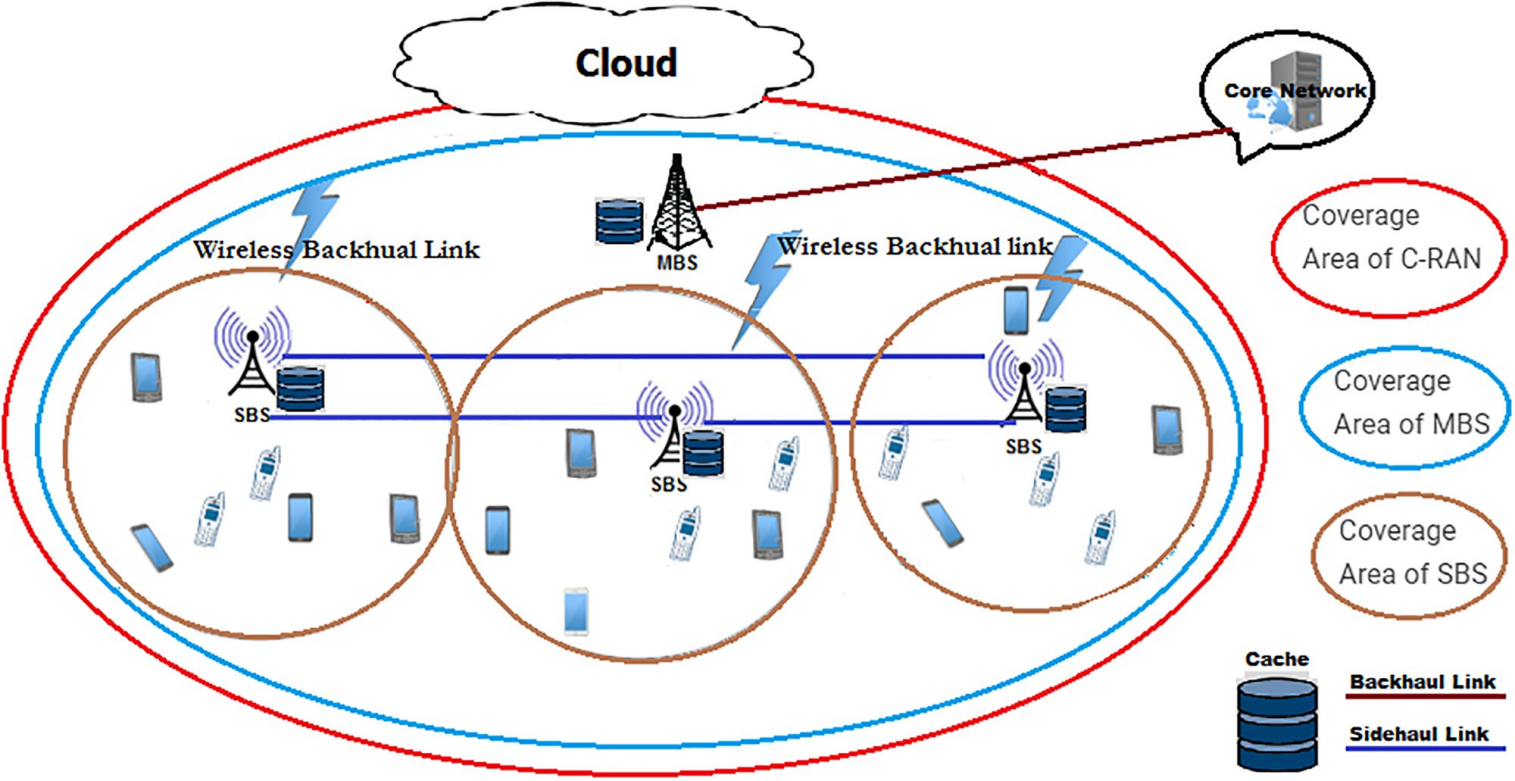
System model of distributed caching in B5G cellular network.

This section gives a detailed description of the system model. The cooperative distributed network with cache-enabled SBSs and MBS along with MSs is considered as shown in Fig 1.

### 3.1 Network model

We consider a cellular network that consists of a cloud, a TDD MBS, *M* TDD SBSs, and *N* MSs as shown in Fig 1. An MBS is a BS of a cellular network, which is donated by G. An MBS is employed to collect the relevant information from all the SBSs in addition to controlling them. The SBSs and MSs are spatially distributed according to two independent homogeneous Poisson Point Process (hPPP) Φ_*B*_ and Φ_*U*_ with the density of SBSs and MSs are represented as λ_*B*_ and λ_*U*_, respectively. The set of SBSs is indicated by $B=\{B_{j}:j=1,2,\ldots ,M\}$ serving a set of MSs $U=\{U_{i}:i=1,2,\ldots ,N\}$. All SBSs are connected to the MBS and a cloud through a backhaul link. While each SBS is bidirectionally connected with its neighboring SBSs via *X*2 interface (a sidehaul wireless link). All SBSs are in an active mode to be associated with at least one MS to serve. Each MS selects its local SBS based on its propagation distance. Therefore, the set of MSs, which serves by an SBS *B*_*j*_ is denoted by *U*_*j*,*i*_: 1 ≤ *i* ≤ *n*, 1 < *j* < *M*, *n* < *N*, where *i* represents an MS served by an SBS *B*_*j*_. In this work, we consider the uplink transmission scenario.

### 3.2 Cache model

We consider *M* + 1 caches across the cellular network. The rest of the characteristics are as under:

#### 3.2.1 Cache storage

The set of SBSs caches is denoted by $∁=\{C_{B_{j}}:j=1,2,\ldots ,M\}$. Each cache stores a set of popular contents denoted by $Ϝ=\{F^{l}:l=1,2,\ldots ,w\}$, where w is the total number of cached contents. The SBS’s cache $C_{B_{j}}$ stores a set of popular contents denoted by $C_{B_{j},f^{l}}=\{C_{B_{j},f^{1}},C_{B_{j},f^{2}},\ldots ,C_{B_{j},f^{l}}:1\leq j\leq M,1\leq l\leq w\}$, where *l* is the total number of the cached contents of *B*_*j*_. The popularity of content ($C_{B_{j},f^{l}}$) is denoted by $\varrho^{C_{B_{j},f^{l}}}$ and is modeled by Zipf distribution according to [38]

$$\begin{array}{c} \varrho^{C_{B_{j},f^{l}}}=\frac{{r^{C_{B_{j},f^{l}}}}^{-\delta }}{\sum_{l=1}^{w}{C_{B_{j},f^{l}}}^{-\delta }}, \end{array}$$ (1)

where $r^{C_{B_{j},f^{l}}}$ represents the popularity rank of the content $C_{B_{j},f^{l}}$, and *δ* is the skewness of the popularity distribution.

Each cached content has some attributes such as name, size, hash key, length, etc. The set of attributes of each cached content is denoted by $P(C_{B_{j},f^{l},\kappa })=\{P(C_{B_{j},f^{l},1}),P(C_{B_{j},f^{l},2}),\ldots ,P(C_{B_{j},f^{l},\kappa })\}$, where *l* is the serial number of content in a cache and *κ* is its maximum number of attributes. The attributes of each cached content will be used for matching to determine the duplicate content and eliminate it (More details in section 4).

The total storage capacity *W* of all *M* + 1 cache in the network can be represented as;

$$\begin{array}{c} W=S\left(G_{∁}\right)+\sum_{j=1}^{M}S\left(C_{B_{j}}\right),1<j\leq M, \end{array}$$ (2)

where $S(G_{∁})$ is the storage capacity of MBS’s cache and $S(C_{B_{j}})$ is the storage capacity of an SBS’s cache.

#### 3.2.2 Distributed caching model

We are considering a cooperative distributed cache, where caches are located at the SBSs and MBS, which appear to be working as a single cache. The contents are stored in a distributed manner across the network. The rationale of this content segmentation is based on [35, 43], where authors argued that the larger size of the contents significantly affects the cache effectiveness due to the scarcity of the storage space.

Therefore, we have proposed that small-sized contents may be stored locally at an SBS, while large size contents can be split into *Q* segments [27, 38, 56].

The set of segments of a content can represented as $\tau_{C_{B_{j},f^{l}}}=\left\{{\left(C_{B_{j},f^{l}}\right)}_{1},{\left(C_{B_{j},f^{l}}\right)}_{2},\ldots ,{\left(C_{B_{j},f^{l}}\right)}_{Q}\right\}$ for all 1 ≤ *l* ≤ *w*, where the size of each segment is determined based on the content’s size ($S(C_{B_{j},f^{l}})$) and the free space of the local SBS and its neighboring. The segment(s) stored in a separate cache create(s) $E_{Q}^{1}$ encoded packets for each segments as $E_{Q}^{1}=\left\{e_{1}^{1},e_{2}^{1},\ldots ,e_{Q}^{1}\right\}$ using MDS code [59], where $e_{1}^{1}$ represents encoded packets for ($C_{B_{j},f^{l}}$)_1_ and so on. Furthermore, if the content is too large, it will be cached at the MBS’s cache as a whole content. The placement of segment(s) is determined in each cache by using the Hash key $H(C_{B_{j},f^{l}})$ of the whole content.

#### 3.2.3 Cache availability and efficiency

Cache availability and efficiency can be evaluated using the cache hit ratio and cache miss ratio. A cache hit occurs, when the content is available in the cache, otherwise, it is a cache miss. According to [60, 61], a cache hit and miss of each SBS *B*_*j*_ are given as:

$$\begin{array}{c} Hit_{r}^{C_{B_{j}}}=\left(\frac{Hit_{n}^{C_{B_{j}}}}{Hit_{n}^{C_{B_{j}}}+Miss_{n}^{C_{B_{j}}}}\right), \end{array}$$ (3)

where $Hit_{n}^{C_{B_{j}}}$ and $Miss_{n}^{C_{B_{j}}}$ are the counter of the cache hit and cache miss of SBS *B*_*j*_, respectively.

$$\begin{array}{c} Miss_{r}^{C_{B_{j}}}=\left(1-Hit_{r}^{C_{B_{j}}}\right). \end{array}$$ (4)

In the scenario of the cache-enabled uplink, the availability of data in a cache is categorized as follows:

1.
$Hit_{n}^{C_{B_{j}}}=1$ : New content is available in distributed cache, therefore, an MS send Message of Target Destination (MoTD)(More details on MoTD in Section(4.2)) rather than actual content.
2.
$Hit_{n}^{C_{B_{j}}}=0$ : New content is unavailable in the distributed cache, therefore, new content will be uploaded.

### 3.3 Communication model

The uplink capacity of an MS *U*_*j*,*i*_ denoted by ${ℜ}_{U_{j,i}}^{UL}$, and can be represented as [62, 63]

$$\begin{array}{c} {ℜ}_{U_{j,i}}^{UL}=B{\text{log}}_{2}\left(1+SINR_{U_{j,i}}\right), \end{array}$$ (5)

where *B* represents the channel bandwidth and $SNR_{U_{j,i}}$ is the signal-interference-to-noise ratio (SINR) of the received signal from MS *U*_*j*,*i*_ at its serving SBS *B*_*j*_ and can be represented as,

$$\begin{array}{c} SINR_{U_{j,i}}=\frac{TP_{U_{j,i}}^{ul}H_{j,i}^{ul}{\left\|d\left(U_{j,i},B_{j}\right)\right\|}^{-\alpha }}{\sum_{i\in I}I_{i}+\sigma^{2}}, \end{array}$$ (6)

where $TP_{U_{j,i}}^{ul}$ is the uplink transmit power of MS *U*_*j*,*i*_. $H_{j,i}^{ul}$ is the corresponding uplink channel gain. ‖.‖ stands for Euclidean norm and *d*(*U*_*j*,*i*_, *B*_*j*_) is the separation distance between *U*_*j*,*i*_ and *B*_*j*_. *α* is the path loss exponent. *I*_*i*_ is a set of interfering MSs. *σ*^2^ is the noise power spectral density at an MS.

Using (5) and (6), the (5) can be rewritten as;

$$\begin{array}{c} {ℜ}_{U_{j,i}}^{UL}=B\;{\text{log}}_{2}\left(1+\frac{TP_{U_{j,i}}^{ul}H_{j,i}^{ul}{\left\|d\left(U_{j,i},B_{j}\right)\right\|}^{-\alpha }}{\sum_{i\in I}I_{i}+\sigma^{2}}\right). \end{array}$$ (7)

Therefore, the uplink network capacity is the sum of cache hit and miss ratios of both the access link (MS) and backhaul (SBS) and is given by:

$$\begin{array}{c} {ℜ}_{Net}^{UL}=(\begin{array}{c} \begin{array}{c} \underset{uplink\;data\;rate\;of\;MS}{\underset{︸}{\sum_{f^{l}\in ,\notin C_{B_{j}}}B\;{\text{log}}_{2}\left(1+\frac{TP_{U_{j,i}}^{ul}H_{U_{j,i}}^{ul}{\left\|d_{U_{j,i},B_{j}}\right\|}^{-\alpha }}{\sum_{i\in I}I_{i}+\sigma^{2}}\right)}}+\\ \underset{uplink\;data\;rate\;of\;SBS}{\underset{︸}{\sum_{f^{l}\notin ,\in G_{C}}B\;{\text{log}}_{2}\left(1+\frac{TP_{B_{j}}^{ul}H_{B_{j}}^{ul}{\left\|d_{B_{j},G}\right\|}^{-\alpha }}{\sigma^{2}}\right)}} \end{array} \end{array}), \end{array}$$ (8)

where $TP_{B_{j}}^{ul}$ is the uplink transmission power of the SBS. $H_{B_{j}}^{ul}$ is the corresponding UL channel gain by an MBS/C-RAN. ‖.‖ stands for Euclidean norm and $d(B_{j},G)$ is the separation distance between *B*_*j*_ and G.

### 3.4 Energy consumption model

In this subsection, we describe the Energy Consumption (EC) of an MS, an SBS, and a cache as follows.

#### 3.4.1 Mobile station energy consumption ($EC_{U_{j,i}}$)

The MS $EC_{U_{j,i}}$ is given according to [33, 64]:

$$\begin{array}{c} EC_{U_{j,i}}=EC_{m}+EC_{op}+ET_{U_{j,i}}^{ul}+EC_{r}, \end{array}$$ (9)

where *EC*_*m*_ and *EC*_*op*_ are energy consumption of performing matching and other operations, respectively. *EC*_*r*_ is energy-consuming for receiving the packet(s) of an SBS’s reply. $ET_{U_{j,i}}^{ul}$ is the energy consumption for transmitting data by the MS. In case of cache hit, $ET_{U_{j,i}}^{ul}$ is only calculated for the communication cost of sending the MoTD message to an SBS, else $ET_{U_{j,i}}^{ul}$ is calculated for the communication cost of sending the whole content.

According to (9), the average EC of the MSs $\left(EC_{U}^{avg}\right)$ is given as,

$$\begin{array}{c} EC_{U}^{avg}=\frac{\sum_{j=1}^{M}\sum_{i=1}^{n}EC_{U_{j,i}}}{N} \end{array}$$ (10)

#### 3.4.2 Caching energy consumption ($C_{B_{j}}^{EC}$)

The caching energy consumption $C_{B_{j}}^{EC}$ is the energy spent by the cache to perform the different operations such as cache hit/miss, cache management, cooperation among distributed cache and is given according to [65] as:

$$\begin{array}{c} \begin{array}{c} C_{B_{j}}^{EC}=Hit_{r}^{C_{B_{j}}}\times EC_{Hit}+Miss_{r}^{C_{B_{j}}}\times EC_{Miss}\;\;\\ EC_{Hit}=EC_{bus}+EC_{cell}\;\;\;\;\;\;\\ EC_{Miss}=EC_{bus}+EC_{cell}+EC_{pad}+EC_{chip}\;\; \end{array}\}, \end{array}$$ (11)

where *EC*_*Hit*_ and *EC*_*Miss*_ are the energy consumption of cache hit and miss, respectively. *EC*_*bus*_, *EC*_*cell*_, *EC*_*pad*_, and *EC*_*chip*_ are energy consumed of address and data bus, sum cell arrays, address and data pads of processor and off-chip cache, respectively. More detail about the calculation of the *EC*_*bus*_, *EC*_*cell*_, *EC*_*pad*_, and *EC*_*chip*_ in [65].

In the case of cooperative distributed caching, an SBS’s cache not only consumes energy for data exchange but also for content partitioning. In addition, according to the proposed framework in Section(4), each SBS generates and broadcasts the list of its cache contents to facilitate content matching. Energy consumption of all these operations is considered to compute *EC*_*CDC*_.

Therefore, (11) can be rewritten as,

$$\begin{array}{c} C_{B_{j}}^{EC}=Hit_{r}^{C_{B_{j}}}\times EC_{Hit}+Miss_{r}^{C_{B_{j}}}\times EC_{Miss}+EC_{CDC}, \end{array}$$ (12)

where the $Miss_{r}^{C_{B_{j}}}$ and $Hit_{r}^{C_{B_{j}}}$ of an SBS *B*_*j*_ are given according to (3) and (4).

#### 3.4.3 SBS energy consumption ($EC_{B_{j}}$)

According to [66], the EC of the SBS is given as

$$\begin{array}{c} \begin{array}{c} P_{comp}=P_{RFC}+P_{BCK}+P_{op_{c}}\;\;\;\;\;\left(a\right)\\ P_{op_{c}}=\sum_{i=1}^{n}{\left(EC_{op_{j}}+ET_{B_{j}}^{ul}+EC_{r_{j}}\right)}_{U_{j,i}}+EC_{e_{B_{j}}}\;\left(b\right) \end{array}\}, \end{array}$$ (13)

where *P*_*comp*_ is the total energy consumption by the following: *P*_*RFC*_, *P*_*BCK*_, and $P_{op_{c}}$ are the energy consumption by radio frequency, backhaul link, and other operations related to the communication and contents, respectively. The serial number of the MS *U*_*j*,*i*_ is represented as ${\left(EC_{op_{j}}+ET_{B_{j}}^{ul}+EC_{r_{j}}\right)}_{U_{j,i}}$ that denoted to the energy consumption of an SBS *B*_*j*_ for executing operations, transmitting and receiving of contents, which are uploaded by the MS *U*_*j*,*i*_, respectively. $ET_{B_{j}}^{ul}$ is the energy consumption for transmitting data by SBS *B*_*j*_. $EC_{e_{B_{j}}}$ is the EC of an SBS *B*_*j*_ for the execution of the cache replacement policy if the cache is full.

In case of a cache hit, $E_{r_{i}}$ is calculated only for the cost of receiving the MoTD at SBS *B*_*j*_, otherwise $E_{r_{i}}$ is calculated for the cost of receiving the whole content.

Using Eqs (11) and (13), we can write;

$$\begin{array}{c} EC_{B_{j}}=P_{comp}+C_{B_{j}}^{EC}. \end{array}$$ (14)

Therefore, the average EC of the SBSs ($EC_{B}^{avg}$) is given as

$$\begin{array}{c} EC_{B}^{avg}=\frac{\sum_{j=1}^{M}EC_{B_{j}}}{M} \end{array}$$ (15)

Finally, according to (10) and (15), the EC of the overall cellular network is given by:

$$\begin{array}{c} EC_{N}=EC_{U}^{avg}+EC_{B}^{avg} \end{array}$$ (16)

### 3.5 Spectrum Efficiency (SE) model

By definition, the uplink SE is determined by the relation between the uplink capacity ℜ^*UL*^ and the total available bandwidth *B* as given by [67],

$$\begin{array}{c} SE=\frac{{ℜ}^{UL}}{B}. \end{array}$$ (17)

According to the Shannon channel capacity, the maximum bit rate is given by

$$\begin{array}{c} {ℜ}^{UL}=B\;{\text{log}}_{2}\left(1+SINR\right). \end{array}$$ (18)

Then, according to (17) and (18), the SE is given as

$$\begin{array}{c} SE=\frac{{ℜ}^{UL}}{B}=\frac{B\;{\text{log}}_{2}\left(1+SINR\right)}{B}={\text{log}}_{2}\left(1+SINR\right). \end{array}$$ (19)

## 4 Proposed framework for distributed uplink cache

**Fig 2:**
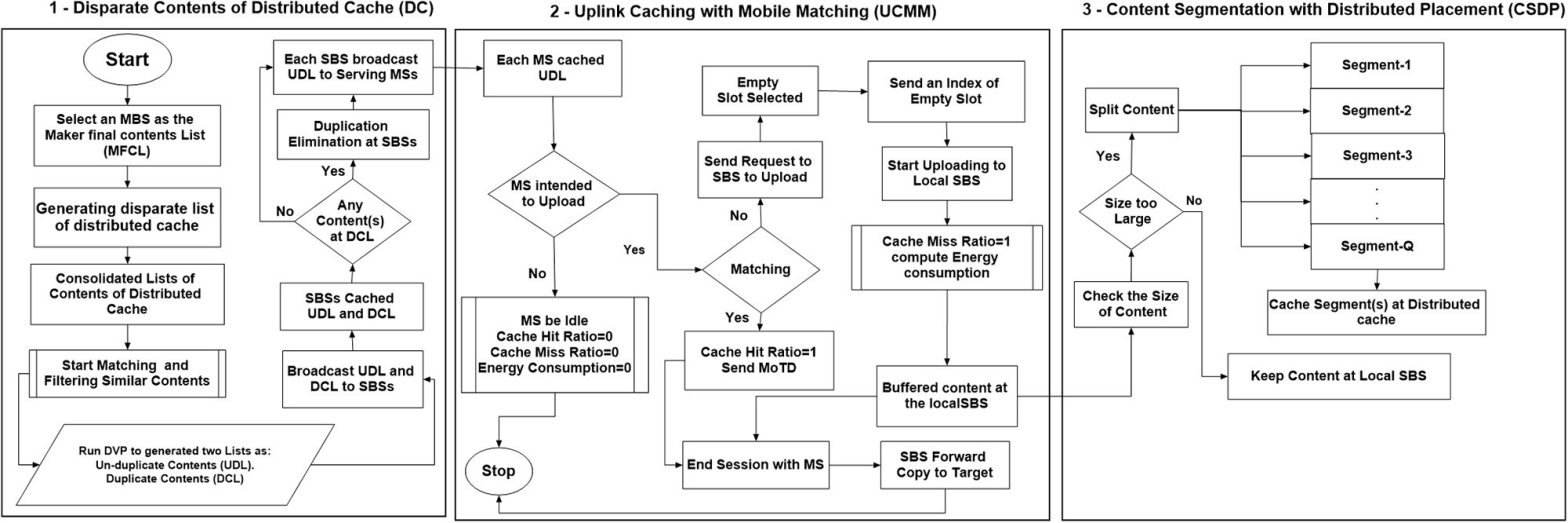
Proposed framework for distributed uplink caching.

In the distributed cache, there are three challenges. Firstly, content duplication among distributed cache. Secondly, an MS’s inability to know about the cache contents. Thirdly, the segmentation and caching distributively the new content with a large size. In order to address these challenges and improve the user’s quality of experience, a framework for distributed uplink caching have proposed. There are three main components of the proposed framework namely, generating a list of cache contents, performing matching at an MS, and content segmentation for distributed cache placement as shown in Fig 2.

### 4.1 Generating disparate list of distributed cache for uplink transmission

**Fig 3:**
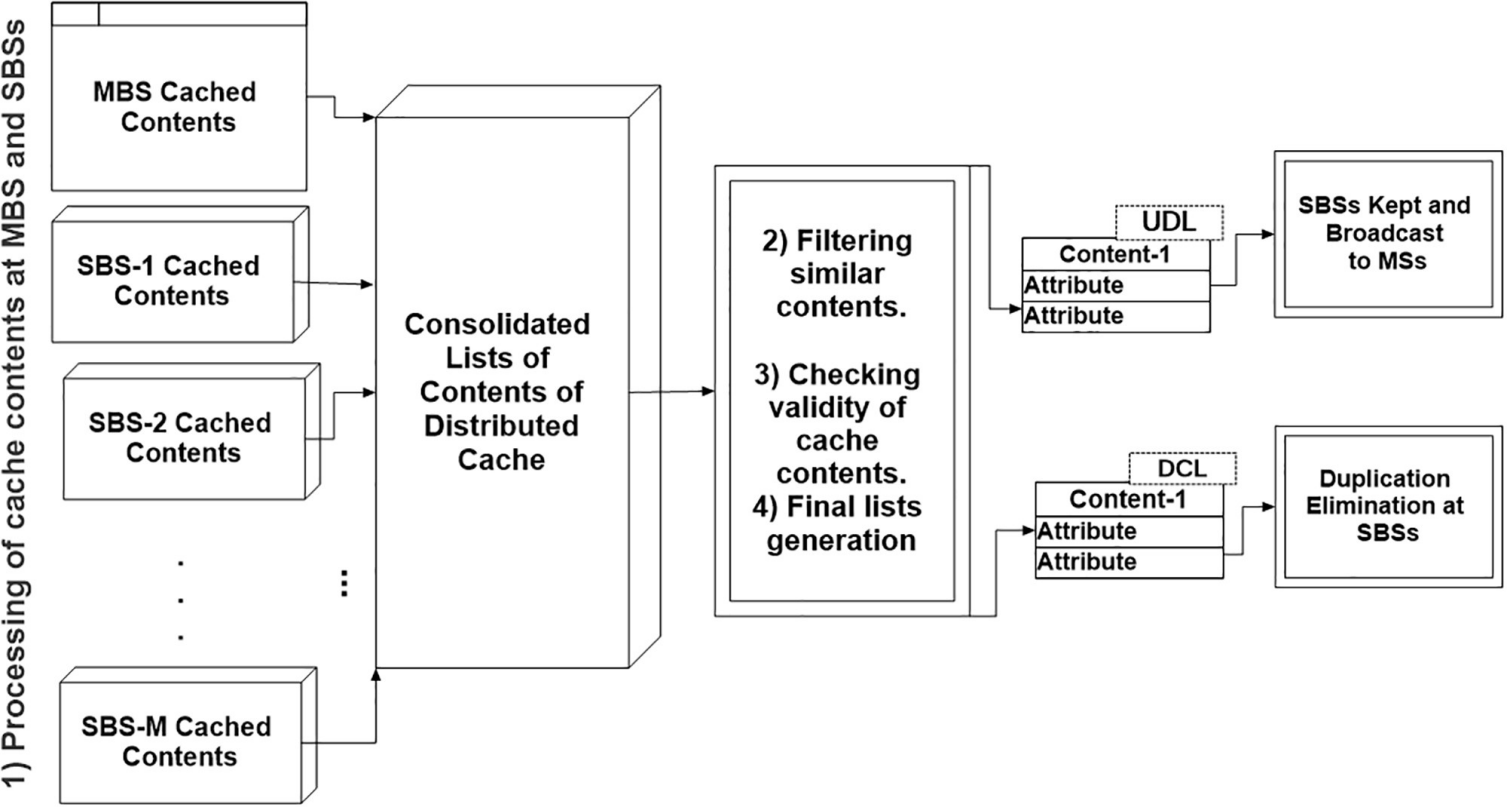
Preparing and generating the final list of cache contents.

It is of paramount importance that an MS has a list of contents that are cached to avoid unnecessary uploads. In this subsection, the algorithm of generating the content list is explained in detail. An MBS is designated as a marker final content list (MFCL). The goal of MFCL is to create two content lists namely, Un-Duplicated content List (UDL) and Duplicated Content List (DCL). As its name implies, UDL consists of un-duplicated cache contents that can be used by an MS for content matching. Whereas, the DCL contains duplicated cache contents, which will be used for evicting identical contents. The rest of this section explains the process of creating these contents lists, which is also shown in Fig 3.

#### 4.1.1 Processing of cache contents at MBS and SBSs

The processing involves creating a list of cached contents at an MBS and all SBSs in the network.

*4.1.1.1 MBS cache contents*. The information about the SBSs that are connected to an MBS can be expressed as;

$$\begin{array}{c} B_{Loc}^{M\times 4}=|\begin{array}{cccc} B_{1}^{ID} & C_{B_{1}}^{ID} & B_{1}^{L} & C_{B_{1}}^{f_{S}}\\ B_{2}^{ID} & C_{B_{2}}^{ID} & B_{2}^{L} & C_{B_{2}}^{f_{S}}\\ \ldots & \ldots & \ldots & \ldots \\ B_{M}^{ID} & C_{B_{M}}^{ID} & B_{M}^{L} & C_{B_{M}}^{f_{S}} \end{array}|, \end{array}$$ (20)

where *M* rows present the total number of the SBSs. The columns present the characteristics of SBS such that $B_{j}^{ID}$ and $C_{B_{j}}^{ID}$ represent ID of an SBS *B*_*j*_ and its cache, respectively. Whereas, $B_{j}^{L}$ is the SBS location in the coverage area of an MBS along with $C_{B_{j}}^{f_{S}}$, which represents the free space of each SBS and is computed as:

$$\begin{array}{c} C_{B_{j}}^{f_{S}}=S\left(C_{B_{j}}\right)-\sum_{l=1}^{w}S\left(C_{B_{j},f^{l}}\right). \end{array}$$ (21)

The list of contents of the MBS’s cache can be expressed as;

$$G_{∁}^{w\times \kappa }=\left|\begin{array}{cccc} P(G_{{∁}_{f^{1}},1}) & P(G_{{∁}_{f^{1}},2}) & \ldots & P(G_{{∁}_{f^{1}},\kappa })\\ P(G_{{∁}_{f^{2}},1}) & P(G_{{∁}_{f^{2}},2}) & \ldots & P(G_{{∁}_{f^{2}},\kappa })\\ \ldots & \ldots & \ldots & \ldots \\ P(G_{{∁}_{f^{w}},1}) & P(G_{{∁}_{f^{w}},2}) & \ldots & P(G_{{∁}_{f^{w}},\kappa }) \end{array}\right|,$$ (22)

where *w* rows present the total number of cached contents while the *κ* is the maximum number of attributes of the content.

*4.1.1.2 SBSs caches contents*. The list of SBS’s cached contents that is denoted by $C_{B_{j}}^{w\times \kappa }$, and can be expressed as;

$$\begin{array}{c} C_{B_{j}}^{w\times \kappa }=|\begin{array}{cccc} P(C_{B_{j},f^{1},1}) & P(C_{B_{j},f^{1},2}) & \ldots & P(C_{B_{j},f^{1},\kappa })\\ P(C_{B_{j},f^{2},1}) & P(C_{B_{j},f^{2},2}) & \ldots & P(C_{B_{j},f^{2},\kappa })\\ \ldots & \ldots & \ldots & \ldots \\ P(C_{B_{j},f^{w},1}) & P(C_{B_{j},f^{w},2}) & \ldots & P(C_{B_{j},f^{w},\kappa }) \end{array}|, \end{array}$$ (23)

where *w* is the total number of each SBS’s cached contents.

Then, the total number of contents in all the SBS’ caches can be represented as

$$\begin{array}{c} C_{B}=\left\{C_{B_{1}}^{w\times \kappa },C_{B_{2}}^{w\times \kappa },\ldots ,C_{B_{M}}^{w\times \kappa }\right\}. \end{array}$$ (24)

*4.1.1.3 Consolidated list of distributed cache contents*. Every SBS sends its cache contents as shown in (23), to an MFCL, which is an MBS acting as a marker. After receiving the caches contents lists from all the SBSs, an MFCL applies row-wise combination function to generate a consolidated list $C_{D_{f}}^{T_{f}\times \kappa }$ as following:

$$\begin{array}{c} C_{D_{f}}^{T_{f}\times \kappa }=|\begin{array}{c} G_{∁}^{w\times \kappa }\\ C_{B_{1}}^{w\times \kappa }\\ C_{B_{2}}^{w\times \kappa }\\ \ldots \\ C_{B_{M}}^{w\times \kappa } \end{array}|, \end{array}$$ (25)

where *T*_*f*_ is the total number of all cached contents across a distributed cache.

#### 4.1.2 Filtering similar contents

The consolidated list generated by an MFCL may have similar contents received from the various SBSs’ cache, in addition to different types of data such as nominal, ordinal, interval, and ratio. In order to remove duplication, the MFCL performs matching among attributes of contents of $C_{D_{f}}^{T_{f}\times \kappa }$ as shown in (25).

In order to compute dissimilarity in (25), we considered typical and target contents denoted by *y* and *y*^•^, respectively. The dissimilarity can be calculated as [68]

$$\begin{array}{c} dissim\left(y,y^{•}\right)=\frac{\sum_{P_{f}=1}^{\kappa }\partial_{y,y^{•}}^{\left(P_{f}\right)}D_{y,y^{•}}^{\left(P_{f}\right)}}{\sum_{P_{f}=1}^{\kappa }\partial_{y,y^{•}}^{\left(P_{f}\right)}}, \end{array}$$ (26)

where *dissim*(*y*, *y*^•^) represents the difference between the typical content *y* and target content *y*^•^, (*P*_*f*_) represents the attributes of content, *κ* is the maximum number of attributes of content. $D_{y,y^{•}}^{\left(P_{f}\right)}$ is the contribution of feature (*P*_*f*_) to the dissimilarity between *y* and *y*^•^. Whereas, $\partial_{y,y^{•}}^{\left(P_{f}\right)}$ is the indicator.

The fetching value principle of the $\partial_{y,y^{•}}^{\left(P_{f}\right)}$: If either $P_{y}^{\left(P_{f}\right)}$ or $P_{y^{•}}^{\left(P_{f}\right)}$ has a missing value of the (*P*_*f*_) attribute or $P_{y}^{\left(P_{f}\right)}=P_{y^{•}}^{\left(P_{f}\right)}=0$ and attribute *P*_*f*_ is asymmetric, then $\partial_{y,y^{•}}^{\left(P_{f}\right)}=0$; otherwise $\partial_{y,y^{•}}^{\left(P_{f}\right)}=1$.

The fetching value principle of the $D_{y,y^{•}}^{\left(P_{f}\right)}$:

1. If the attribute *P*_*f*_ of contents is numerical, interval or ratio type, then
  $$\begin{array}{c} D_{y,y^{•}}^{\left(P_{f}\right)}=\frac{|D_{y,P_{f}}-D_{y^{•},P_{f}}|}{max_{h}D_{y,P_{f}}-min_{h}D_{y^{•},P_{f}}}, \end{array}$$ (27)
  where *h* can take all the non-missing value of the attribute *P*_*f*_ of content.
2. If the attribute *P*_*f*_ of contents is nominal or binary, then
  $$\begin{array}{c} D_{y,y^{•}}^{\left(P_{f}\right)}=\{\begin{array}{cc} 0, & D_{y,P_{f}}=D_{y^{•},P_{f}}\\ 1, & D_{y,P_{f}}\neq D_{y^{•},P_{f}} \end{array}. \end{array}$$ (28)
3. If the attribute *P*_*f*_ of contents is ordinal, then calculate ranking $r_{y,P_{f}}$, then
  $$\begin{array}{c} z_{y,P_{f}}=\frac{\left(r_{y,P_{f}}-1\right)}{\left(m_{P_{f}}-1\right)}, \end{array}$$ (29)
  where $r_{y,P_{f}}$ represents the ranking of state in the attribute *P*_*f*_ of contents, $m_{P_{f}}$ is the number of ordered states of *P*_*f*_, and treat $z_{y,P_{f}}$ as a numerical type.

The similarity between *y* and *y*^•^ is computed by:

$$\begin{array}{c} sim\left(y,y^{•}\right)=1-dissim\left(y,y^{•}\right), \end{array}$$ (30)

where *sim*(*y*, *y*^•^) shows similarity of contents (*y*, *y*^•^).

The list of the values of similarity is denoted by $SIM^{T_{f}\times \kappa }$ and can be expressed as;

$$\begin{array}{c} SIM^{T_{f}\times \kappa }=|\begin{array}{ccccc} 1 & s_{1,2} & s_{1,3} & \ldots & s_{1,\kappa }\\ s_{2,1} & 1 & s_{2,3} & \ldots & s_{2,\kappa }\\ s_{3,1} & s_{3,2} & 1 & \ldots & s_{3,\kappa }\\ \ldots & \ldots & \ldots & \ddots & \ldots \\ s_{T_{f},1} & s_{T_{f},2} & s_{T_{f},2} & \ldots & 1 \end{array}|. \end{array}$$ (31)

The closest similar two contents is a column with a maximum value (value 1) among the values across each content. Alternatively, similarity can also be determined by setting a threshold value $sim_{th}^{sh}$ such that

$$\begin{array}{c} sim=\{\begin{array}{cc} similar, & sim\left(y,y^{•}\right)>=sim_{th}^{sh}\\ dissimilar, & sim\left(y,y^{•}\right)<sim_{th}^{sh} \end{array}. \end{array}$$ (32)

In this way, duplicate contents can be identified residing in different caches at SBSs, which can then be subsequently evicted to make space for new content.

#### 4.1.3 Checking validity of cache contents

After identifying similar contents, it is also important to identify the validity of the existing cache contents by the MFCL. The same content in the different cache should be evicted and kept only at one cache. To do this, we proposed Dynamic Validity Period (DVP) with the aim to compute the probability of either keeping or evicting the content from any of the distributed cache. It can be expressed as;

$$\begin{array}{c} DVP^{C_{B_{j},f^{l}}}=RE_{B_{j}}+C_{B_{j}}^{f_{S}}+U_{j,i}+\varrho^{C_{B_{j},f^{l}}}+TimEC_{r}^{C_{B_{j},f^{l}}}, \end{array}$$ (33)

where $RE_{B_{j}}$ is the remaining energy of an SBS *B*_*j*_. $C_{B_{j}}^{f_{S}}$ is the cache free space of an SBS as mentioned in (20) according to (21). *U*_*j*,*i*_ is the total number of the MSs served by the SBS *B*_*j*_. $\varrho^{C_{B_{j},f^{l}}}$ is the popularity of each cached content according to (1). $TimEC_{r}^{C_{B_{j},f^{l}}}$ is the remaining time of content in the cache and is given by:

$$\begin{array}{c} TimEC_{r}^{C_{B_{j},f^{l}}}=Expire_{time}^{C_{B_{j},f^{l}}}-Time_{last_{Hit}}^{C_{B_{j},f^{l}}}, \end{array}$$ (34)

where $Expire_{time}^{C_{B_{j},f^{l}}}$ is the expire time of that content and the $Time_{last_{Hit}}^{C_{B_{j},f^{l}}}$ is the time of the last hit of that content.

The DVP is executed after performing the content similarity and determining the duplicate contents of the distributed cache. Let

$$\begin{array}{c} K=\text{max}(DVP^{C_{B_{1},f^{l}}},\ldots ,DVP^{C_{B_{m},f^{l}}}), \end{array}$$ (35)

where 1 ≤ *m* ≤ *M*. *K* represented the hash key of the contents of DVP ($DVP^{C_{B_{j},f^{l}}}$). It mean is represents all the attributes of the contents. That has highest validity. Therefore, it should be placed in UCL. All the remaining contents will be placed in DCL. This can formally be seen in (38) and (39).

#### 4.1.4 Final list generation

Now that the similarity and validity of the contents are determined, the final lists of duplicated and unduplicated contents can be generated. The *UCL* is the list of un-duplicate contents of the distributed cache, which can be used by an MS as a map to determine whether to upload contents or send a MoTD instead of uploaded the actual content. The UCL can be formed using (25) and (35). The value of *K* in (35) is used to select the content of $C_{D_{f}}^{T_{f}\times \kappa }$ in (25) using the hash key $H(C_{B_{j},f^{l}})$. *K* represents a row in $C_{D_{f}}^{T_{f}\times \kappa }$ in (25), which has maximum validity (DVP). That is indicated by $C_{D_{f}}^{T_{f}\times \kappa }\left(l,*\right)$, and can be presented as

$$\begin{array}{c} C_{D_{f}}^{T_{f}\times \kappa }\left(l,*\right)=|\begin{array}{cccc} P(C_{B_{j},f^{l,*},l}) & P(C_{B_{j},f^{l,*},2}) & \ldots & P(C_{B_{j},f^{l,*},\kappa }) \end{array}|, \end{array}$$ (36)

where *l* is the serial number of the row with max DVP such that *l* ∈ [1, *T*_*f*_].

$C_{D_{f}}^{T_{f}\times \kappa }\left(f,*\right)$ represents the row of the matrix in (25), which has maximum validity as shown in (35). Eq (36) shows max value of DVP of a single content. The same process is performed for all the contents of distributed cache. It will provide max values of DVP of all the duplicate contents. It can be seen as a cluster of duplicated contents with different DVP values. The contents with maximum DVP value will be placed in UCL, while the remaining values will be placed in DCL. Based on this discussion, UCL can be represented as

$$\begin{array}{c} UCL^{T\times \kappa }=UCL^{T\times \kappa }+C_{D_{f}}^{T_{f}\times \kappa }\left(l,*\right), \end{array}$$ (37)

where *l* ∈ [1, *T*_*f*_] represent the rows with max DVP values. When all the rows with max DVP are added the final UCL can be expressed as;

$$\begin{array}{c} UCL^{T\times \kappa }=|\begin{array}{cccc} P_{C_{f_{1,1}}} & P_{C_{f_{1,2}}} & \ldots & P_{C_{f_{1,\kappa }}}\\ P_{C_{f_{2,1}}} & P_{C_{f_{2,2}}} & \ldots & P_{C_{f_{2,\kappa }}}\\ \ldots & \ldots & \ldots & \ldots \\ P_{C_{f_{T,1}}} & P_{C_{f_{T,2}}} & \ldots & P_{C_{f_{T,\kappa }}} \end{array}|. \end{array}$$ (38)

where *T* ≤ *T*_*f*_ presents the total number of unduplicated contents among all distributed cache.

The DCL is the list of the duplicated contents of the distributed cache, which determines the contents to be evicted from SBS(s) caches. As previously mentioned that the max DVP values of contents are placed in UCL, the remaining duplicated contents are added to DCL and can be expressed as;

$$\begin{array}{c} DCL^{T^{•}\times \kappa }=|\begin{array}{cc} DVP^{C_{B_{j},f^{1}}} & H(C_{\left(B_{j}/G\right),f^{l}})\\ DVP^{C_{B_{j},f^{2}}} & H(C_{\left(B_{j}/G\right),f^{2}})\\ \ldots & \ldots \\ DVP^{C_{B_{j},f^{T^{•}}}} & H(C_{\left(B_{j}/G\right),f^{T^{•}}}) \end{array}|. \end{array}$$ (39)

where *T*^•^ is the total number of the duplicated contents across a distributed cache to be eliminated from their caches.

#### 4.1.5 Duplication elimination at sbs and broadcast to the MSs

After removing duplicate contents from MBS’s cache, UCL and DCL are sent to all the SBSs. The elimination of the duplicate contents as suggested by MFCL is the result of *DVP* in the DCL as shown in (39). The contents with low *DVP* are evicted from their caches and can be represented as $victim(DVP^{C_{B_{j},f^{l}}},C_{B_{j}},\bar{Time})$, where $\bar{Time}$ is the time of evicted content. The evicted contents are subsequently reported to MBS for updating $B_{Loc}^{M\times 4}$ as shown in (20).

Additionally, each SBS *B*_*j*_ broadcasts the UCL to all its serving MSs *U*_*j*,*i*_, which can be used for matching, subsequently. The contents of the distributed cache are updated continuously, and their popularity is getting changed for reasons such as times of uploads, downloads, shares, views, etc. Therefore, UCL is updated and broadcast to all the MSs at a periodic time to ensure content consistency among the distributed cache and the MSs.

#### 4.1.6 Proposed (*DC*)^2^ scheme

Based on the discussion, an algorithm is proposed, which is called *Disparate Content of Distributed Cache* (*DC*)^2^ as shown in Algorithm 1. Each previous section from (4.1.1) to (4.1.5) corresponds to a step of the proposed algorithm. The steps of the proposed algorithm can be summarized as follows: The proposed (*DC*)^2^ algorithm consists of 5 major steps. In step 1, all the caches at SBSs and MBS are processed followed by a similarity check in step 2. The proposed algorithm further checks the validity of the contents before generating the final lists in step 3 and 4, respectively. Finally, duplicate contents are removed from all the target SBSs and the un-duplicated list is sent to all the MSs to perform content matching in step 5.

**Algorithm 1**: Disparate Contents of Distributed Cache (*DC*)^2^

1 **Input**: $B_{Loc}^{M\times 3}$, $G_{∁}^{w\times \kappa }$, B, $C_{B}=\left\{C_{B_{1}}^{w\times \kappa },C_{B_{2}}^{w\times \kappa },\ldots ,C_{B_{M}}^{w\times \kappa }\right\}$, *U*_*j*,*i*_.

2 **Output**: UCL, DCL.

3 **Step 1**: **Processing of cache contents at SBSs and MBS**

4 *T*_*f*_,*T*,*T*^•^←0, Counters of all, unduplicated and duplicated contents; $Sim_{th}^{sh}$ ← the threshold of similarity;

5 Create $C_{D_{f}}^{T_{f}\times \kappa }$ by adding $G_{∁}^{w\times \kappa }$;

6 **for** (*j* = 1, *j* ≤ *M*, *j*++) **do**

7  **for** (*l* = 1, *l* ≤ *w*, *l*++) **do**

8   Create new row in $C_{D_{f}}^{T_{f}\times \kappa }$;

9   Add $C_{B_{j},f^{l}}$ and its Attributes $P(C_{B_{j},f^{l},k})$ to $C_{D_{f}}^{T_{f}\times \kappa }$ (25);

10   *T*_*f*_++;

11 MFCL created $C_{D_{f}}^{T_{f}\times \kappa }$ (25) ← list of all distributed cached contents;

12 **Step 2**: **Filtering similar contents**

13 **for** (*y* = 1, *y* ≤ *T*_*f*_, *y*++) **do**

14  $D_{f}^{\bar{t}}$ ← Temporary list of duplicated contents;

15  **for** (*y*^•^ = *y* + 1, *y*^•^ ≤ *T*_*f*_, *y*^•^++) **do**

16   *dissim*(*y*, *y*^•^) ← 0;

17   Clear *D*^(*T*+1)×*K*^;

18   **for** (*q* = 1, *q* ≤ *κ*, *q*++) **do**

19    **if** ($P_{y}^{\left(P_{f}\right)}$ ∣∣ $P_{y^{•}}^{\left(P_{f}\right)}$ = *Null*) ∣∣ ($P_{y}^{\left(P_{f}\right)}$ ∣∣ $P_{y^{•}}^{\left(P_{f}\right)}$ = *0*) & (*P*_*f*_ *is Asymmetric*) **then**

20     $\delta_{y,y^{•}}^{\left(P_{f}\right)}=0$

21    **else**

22     $\delta_{y,y^{•}}^{\left(P_{f}\right)}=1$

23    **if** (*Numeric* —— *Interval* —— *Ratio Attributes*) **then**

24     $D_{y,y^{•}}^{\left(P_{f}\right)}$ ← according to (27);

25    **if** (*Nominal Attributes*) **then**

26     $D_{y,y^{•}}^{\left(P_{f}\right)}$ ← according to (28);

27    **if** (*Ordinal Attributes*) **then**

28     $z_{y,P_{f}}$ ← according to (29);

29    Add $D_{y,y^{•}}^{\left(P_{f}\right)}$ ⇒ *Dissim*(*y*, *y*^•^);

30   Compute Dissimilarity *dissim*(*y*, *y*^•^) according to (26);

31   *sim*(*y*, *y*^•^) ← Calculate Similarity according to (30);

32   Add *sim*(*y*, *y*^•^) ⇒ $SIM^{T_{f}\times \kappa }$ (31);

33   **Step 3**: **Checking validity of cache contents**

34   **if** (*sim*(*y*, *y*^•^)$¿=sim_{th}^{sh}$) **then**

35    $DVP^{C_{D_{y}}^{T_{f}\times \kappa }}$ ← Compute DVP according to (33);

36    Add $D_{y}^{T_{f}\times \kappa }$ to $D_{f}^{\bar{t}}$;

37    $DVP^{C_{D_{y^{•}}}^{T_{f}\times \kappa }}$ ← Compute DVP according to (33);

38    Add $D_{y^{•}}^{T_{f}\times \kappa }$ to $D_{f}^{\bar{t}}$;

39   **Step 4**: **Final List Generation**

40   Add Max $\left(D_{f}^{\bar{t}}\right)$ ⇒ *UCL*^*T*×*κ*^ (38);

41   *T*++;

42   Add others $\left(D_{f}^{\bar{t}}\right)$ ⇒ $DCL^{T^{•}\times \kappa }$ (39);

43   *T*^•^ = *T*^•^ + Count($D_{f}^{\bar{t}}$)—1;

44   Clear $D_{f}^{\bar{t}}$;

45 **Step 5**: **Duplication elimination at SBSs and Broadcast to the MSs**.

46 **for** (*l* = 1, *l* ≤ *T*^•^, *l*++) **do**

47  **for** (*j* = 1, *j* ≤ *M*, *j*++) **do**

48   **if** $H(DCL^{T^{•}\times \kappa }{)}_{f^{l}}$ ∈ ($C_{B_{j}}^{w\times \kappa }$) **then**

49    Remove ($C_{B_{j},f^{l}}$) (as $victim(DVP^{C_{B_{j},f^{l}}},C_{B_{j}},\bar{Time})$);

50 Each SBS *B*_*j*_ broadcast UCL to all serving MSs *U*_*j*,*i*_.

51 **Return** *UCL* & *DCL*.

### 4.2 Cache assist uplink with an MS enabled matching

This section describes the mechanism of performing content matching at an MS after it receives UCL. The process of matching is performed in three steps as described below. In first step, an MS intends to upload new content to its serving SBS *B*_*j*_. It will make a consolidated list comprising of attributes of new contents and UCL. In secon step, an MS will perform matching to check if its available in the cache. Lastly, the contents will only be uploaded in case cache miss i.e. unavailability of the contents in the cache. If the content is enlisted in UCL, an MS will not upload the content. Instead, MoTD will be sent to its serving SBS. It is worth mentioning that if matching is performed at an MS, it will significantly improve the spectrum and energy efficiency. The aforementioned steps are described in details as following.

#### 4.2.1 Consolidated list of attributes of new content and UCL

This subsection provide details of constructing a consolidated list of attributes of new contents and the existing UCL. The main steps are as following.

1. Firstly, an MS creates a list comprising new content and its attributes as shown in (40).
  $$\begin{array}{c} R_{f}^{1\times \kappa }=|\begin{array}{cccc} P\left(R_{j,i}^{f^{1}},1\right) & P\left(R_{j,i}^{f^{1}},2\right) & \ldots & P\left(R_{j,i}^{f^{1}},\kappa \right) \end{array}|, \end{array}$$ (40)
  where $P\left(R_{j,i}^{f^{1}},\kappa \right)$ represents the new content attributes.
2. The unduplicated contents of the distributed cache is shown in (38), which is combined with (40) by using a row-wise combination function to generate a consolidated list. The consolidate list is denoted by *O*^(*T*+1)×*K*^ and is given by;
  $$\begin{array}{c} O^{(T+1)\times K}=|\begin{array}{cccc} P_{C_{f_{1,1}}} & P_{C_{f_{1,2}}} & \ldots & P_{C_{f_{1,\kappa }}}\\ P_{C_{f_{2,1}}} & P_{C_{f_{2,2}}} & \ldots & P_{C_{f_{2,\kappa }}}\\ \ldots & \ldots & \ldots & \ldots \\ P_{C_{f_{T,1}}} & P_{C_{f_{T,2}}} & \ldots & P_{C_{f_{T,\kappa }}}\\ P\left(R_{j,i}^{f^{1}},1\right) & P\left(R_{j,i}^{f^{1}},2\right) & \ldots & P\left(R_{j,i}^{f^{1}},\kappa \right) \end{array}|. \end{array}$$ (41)
  The last row in (41) is the values of attributes of new content.

#### 4.2.2 Matching the attributes of new content and UCL

After constructing the consolidated list, an MS will start matching to check whether the new content is available in the cache. Using (41), the matching among the attributes of new content and the contents in a distributed cache is performed by using the similar process as explained in Section (4.1.2), especially Eqs (26)–(30).

According to [69], the matching is performed between the control item and treat item. In this work, the control item is the UCL, while the new content is a target content. Then, the matching will result in a dissimilarity matrix as shown in (42).

$$\begin{array}{c} D^{(T+1)\times K}=|\begin{array}{cccc} 0 & & & \\ D(C_{f_{1}},C_{f_{2}}) & 0 & & \\ D(C_{f_{1}},C_{f_{3}}) & D(C_{f_{2}},C_{f_{3}}) & 0 & \\ \ldots & \ldots & \ldots & \ldots \\ D(C_{f_{1}},R_{j,i}^{f^{1}}) & D(C_{f_{2}},R_{j,i}^{f^{1}}) & D(C_{f_{T+1}},R_{j,i}^{f^{1}}) & 0 \end{array}|. \end{array}$$ (42)

The last row of (42) shows the dissimilarity between the new and cached content, which can be used to either upload or discard the new contents.

#### 4.2.3 Uploading dissimilar content and ignore similar content

After matching is performed, the content can either be uploaded or an MoTD is sent. If a match is found, the content is not uploaded and an MoTD is sent instead of the actual content. The content will be uploaded, if the match is not found. In other words, contents will only be uploaded in case of a cache miss.

#### 4.2.4 Proposed UCMM scheme

Based on the discussion, an algorithm is proposed, which is called is called an Uplink Caching with Mobile Matching (UCMM) scheme as shown in Algorithm 2 and Fig 2-(Part-2). The proposed UCMM performs matching among the attributes of new contents and UCL at an MSs level to determine if new content is already in a cache, which will eliminate the need of uploading duplicate contents. Each previous section from (4.2.1) to (4.2.3) corresponds to a step of the proposed algorithm. The steps of the proposed algorithm can be summarized as follows: In step 1, consolidate the lists of the attributes of the new content and UCl contents into a list followed by a similarity check in step 2. Finally, decides whether to upload dissimilar content or ignore similar content by sending a MoTD to the serving SBS in step 3.

**Algorithm 2**: Uplink Caching with Mobile Matching (UCMM)

1 **Input**: *UCL*^*T*×*κ*^, $R_{f}^{1\times \kappa }$.

2 **Output**: Matching factor *Matching*_*R*_, Miss/Hit factor *Miss*_*n*_ or *Hit*_*n*_.

3 *Miss*_*n*_ ←0, *Hit*_*n*_ ←0, *Matching*_*R*_ ← false, $sim_{th}^{sh}$ ← Value of threshold of Similarity;

4 *D*^(*T*+1)×*K*^ ← Dissimilarity matrix(42);

5 **Step-1**: **Consolidated List of Attributes of New Content and UCL**.

6 Create $R_{f}^{1\times \kappa }$;

7 Get *UCL*^*T*×*κ*^;

8 Create *O*^(*T*+1)×*K*^ refers(41) by consolidated (38 and 40);

9 **Step-2**: **Matching the Attributes of New Content and UCL**.

10 **for** (*y* = 1, *y* ≤ (*T* + 1), *y*++) **do**

11  $dissim(C_{f_{T,\kappa }},R_{j,i}^{f^{1}})$ ← 0;

12  Clear *D*^(*T*+1)×*K*^;

13  **for** (*q* = 1, *q* ≤ *κ*, *q*++) **do**

14   **if** ($P_{y}^{\left(P_{f}\right)}$ ∣∣ $P_{y^{•}}^{\left(P_{f}\right)}$ = *Null*) ∣∣ ($P_{y}^{\left(P_{f}\right)}$ ∣∣ $P_{y^{•}}^{\left(P_{f}\right)}$ = *0*) & (*P*_*f*_ *is Asymmetric*) **then**

15    $\delta_{y,y^{•}}^{\left(P_{f}\right)}=0$

16   **else**

17    $\delta_{y,y^{•}}^{\left(P_{f}\right)}=1$

18   **if** (*Numeric* —— *Interval* —— *Ratio Attributes*) **then**

19    $D_{y,y^{•}}^{\left(P_{f}\right)}$ ← according to (27);

20   **if** (*Nominal Attributes*) **then**

21    $D_{y,y^{•}}^{\left(P_{f}\right)}$ ← according to (28);

22   **if** (*Ordinal Attributes*) **then**

23    $D_{y,y^{•}}^{\left(P_{f}\right)}$ ← according to (29);

24   Add $D_{y,y^{•}}^{\left(P_{f}\right)}$ ⇒ $dissim(C_{f_{T,\kappa }},R_{j,i}^{f^{1}})$;

25  Compute Dissimilarity $dissim(C_{f_{T,\kappa }},R_{j,i}^{f^{1}})$ according to (26);

26  *D*^(*T*+1)×*K*^ created;

27  Determine value of $sim_{th}^{sh}$;

28  Compute Similarity $sim(C_{f_{T,\kappa }},R_{j,i}^{f^{1}})$ according to (30);

29  **if** ($sim(C_{f_{T,\kappa }},R_{j,i}^{f^{1}})$) ⩾ $sim_{th}^{sh}$) **then**

30   *Matching*_*R*_ ← true;

31   set y← *T* + 1;

32 **Step-3**: **Uploading Dissimilar Content and Ignore Similar content**.

33 **if** (*Matching*_*R*_ = *true*) **then**

34  *Hit*_*n*_+=1;

35  Send MoTD;

36 **else**

37  *Miss*_*n*_+=1;

38  An MS send request to upload the new content;

39  An MS received the $index_{R_{j,i}^{f^{1}}}$;

40  Start uploading $R_{j,i}^{f^{1}}$;

41 Return *Matching*_*R*_, *Miss*_*n*_/*Hit*_*n*_.

### 4.3 Content segmentation and distributed placement

**Fig 4:**
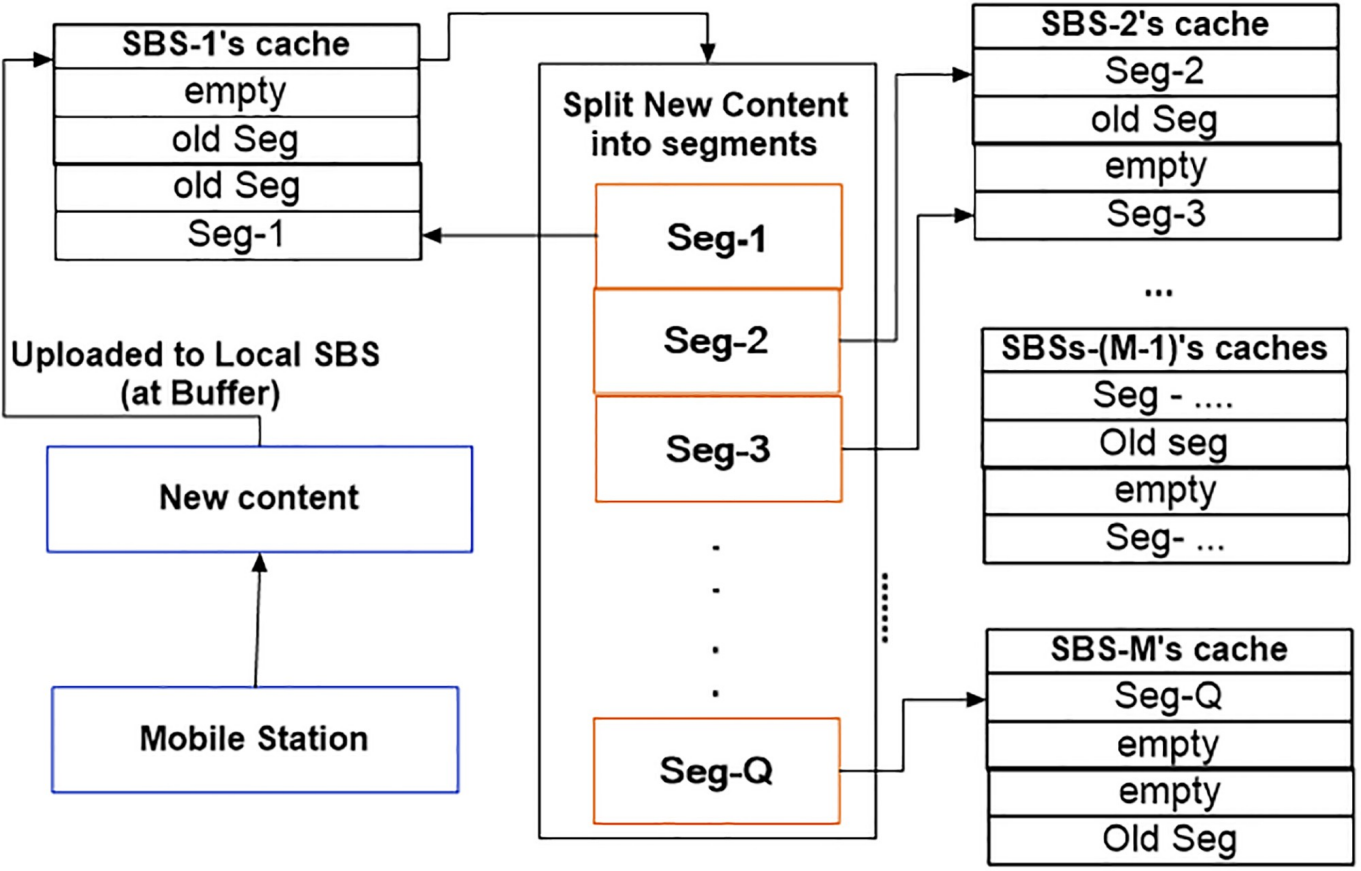
Segmentation of new content across distributed cache.

In the case of distributed caching, the size of contents is a major limiting factor towards the cache effectiveness [27, 38, 56]. The new content size may be too large to accommodate in a single cache, efficiently. Therefore, it is recommended that the new content should be split into smaller segments with same size to be stored distributively. In this vein, we proposed a scheme that advocates to divide the new content into *Q* different segments as a function of new content size $S(R_{j,i}^{f^{1}})$ and available cache space of the corresponding and its neighboring SBSs as shown in Fig 2-(Part-3) and (Fig 4). The rationale of this content segmentation is the effective placement due to the smaller size in distributed cache across multiple SBSs. According to [56], the *Q* is equal $Q\leq (1+count(B_{j^{•}}))$, where 1 is the local SBS *B*_*j*_ and *count*($B_{j^{•}}$) is the number of neighboring SBSs, which have a non-void intersection with $B_{j^{•}}$ and enough free space to cache at least a segment as $B_{j^{•}}≜\left\{B_{j^{•}}\in B_{Loc}^{M\times 4}:C_{B_{j^{•}}}^{f_{S}}>S\left(R_{j,i}^{f^{1}}\right)\right\}$.

In order to facilitate its storage distributively, the segment(s) will be cached in a target distributed cache based on its free space according to (20) and (21). An MDS code method is used to encode the new content into packets as mentioned in Section (3.2.2).

The result of the above discussions is programmatically extracted in a new proposed scheme, which is called *Content Segmentation with Distributed Placement (CSDP)* as shown in Algorithm-3, and in Fig 2-(part-3). The CSDP consists of 2 major steps, where step 1 performs preparation of the required information based on the size and hash key of the new content. In addition, free space of the local and its neighboring SBSs is also considered. Step 2, corresponds to new content splitting into *Q* segments, encoding, and cache placement of segmented contents in a distributed manner.

**Algorithm 3**: Content Segmentation with Distributed Placement (CSDP) Scheme

1 **Input**: $H(R_{j,i}^{f^{1}})$, $S(R_{j,i}^{f^{1}})$, *SBS*_*j*_, $R_{B_{j}}$.

2 **Output**: $E_{Q}^{l}$.

3 **Step 1**: **Preparing the require information**.

4 $S(R_{j,i}^{f^{1}})$: The size of the new content;

5 $H(R_{j,i}^{f^{1}})$: The hash key of the new content;

6 Get the free space of the target SBSs according to (20 and 21);

7 $B^{•}≜\left\{B_{j^{•}}\in B_{Loc}^{M\times 4}:C_{B_{j^{•}}}^{f_{S}}>S\left(R_{j,i}^{f^{1}}\right)\right\}$// Set the target SBSs;

8 **Step 2**: **Split new content and cached it distributively**

9 $Q=1+count(B_{j^{•}}))$ // total number of segments;

10 **for** (*q = 1*, *q¡=Q*, *q*++) **do**

11  Check the $C_{B_{j}}^{f_{S}}$ according to (20 and 21);

12  **if** (*q = 1*) & ($C_{B_{j}}^{f_{S}}>S\left(R_{j,i}^{f^{1}}\right)$) **then**

13   Store *Seg*_*q*_ or set of *Seg*_*q*_s in *B*_*j*_;

14   Encode $e_{q_{j}}^{l}$ using MDS code, $e_{q_{j}}^{l}$ → $e_{R_{f_{q}}}^{B_{j}}$;

15  **else**

16   Select $B_{j^{•}}$ from $B^{•}$;

17   Check $C_{B_{j^{•}}}^{f_{S}}$ of neighboring SBSs according to (20 and 21);

18  **if** ($C_{B_{j^{•}}}^{f_{S}}>S\left(R_{j,i}^{f^{1}}\right)$) **then**

19   Store *Seg*_*q*_ or set of *Seg*_*q*_s in $B_{j^{•}}$;

20   Encode $e_{q_{j^{•}}}^{l}$ using MDS code, $e_{q_{j^{•}}}^{l}$ → $e_{R_{f_{q}}}^{B_{j^{•}}}$;

21 Create the map of the set encoded packets: $E_{Q}^{l}=\left\{e_{1}^{l},e_{2}^{l},\ldots ,e_{Q}^{l}\right\}$;

22 Change the status of new content to be a cached content;

23 **Return** $E_{Q}^{l}$.

### 4.4 Complexity analysis of the proposed framework

As mentioned, and discussed in the previous sections, *M* is the total number of distributed cache. Each cache store *w* contents with a total of *T*_*f*_ contents in the whole distributed network. Each content has *κ* attributes. Additionally, the unduplicated and duplicated contents among distributed cache are *T*, and *T*^•^, respectively. As well, the proposed framework consists of three parts as follows: (*CD*)^2^, *UCMM*, and *CSDP*.

The overall complexity of the proposed framework is (*O*((*M*.*w*)^2^) + *O*(*M*.*w*) + *O*(*M*)), which is computed for the three parts as follows:

#### 4.4.1 Complexity of *CD*^2^ algorithm

The *CD*^2^ is performed to solve the issue of eliminating the duplicated contents among the distributed cache as shown in algorithm 1, which is executed by many operations. The main operations of the iterative have complexity as the following:

1. The operation of the processing of the cache contents at the distributed cache has complexity *O*(*M*.*w*).
2. The operation of filtering similar contents by performing the matching among the attributes of the contents has complexity $O({T_{f}}^{2})$.
3. The eliminating of the duplicate contents from the target distributed cache has complexity *O*(*T*^•^).
4. Finally, the overall complexity of the *CD*^2^ is $O\left(M.w\right)+O\left({T_{f}}^{2}\right)+O\left(T^{•}\right)$, where *T*_*f*_ = (*M*.*w*) but $O\left({T_{f}}^{2}\right)>O\left(T^{•}\right)$ and $O\left({T_{f}}^{2}\right)>O\left(m.w\right)$. Then, the overall complexity of the proposed framework is $O\left({T_{f}}^{2}\right)=O\left({\left(M.w\right)}^{2}\right)$.

#### 4.4.2 Complexity of *UCMM* algorithm

The solution to the cache uplink problem without duplication of the mobile data to be uploaded is solved by algorithm 2 called UCMM. The overall complexity of *UCMM* is *O*(1) in the best case, while *O*(*M*.*w*) in the worst case.

#### 4.4.3 Complexity of *CSDP* algorithm

The solution of the segmentation of the new content with a large size and cache distributively is solved by algorithm 3 called SCDP. The new content will be segmented into *Q* segments with a smaller size to be cached distributively in the target caches, which is equal to or smaller than *M*. The segmentation of new content has complexity *O*(*Q*). While the caching segments of each content have complexity *O*(*M*). Therefore, the overall complexity of the *CSDP* is *O*(*M*).

## 5 Experimental design and evaluation

We have compared our *proposed framework* in different scenarios such as uplink with no-caching *(No-cache)*, cache assisted uplink *(Each-cache)* [33] and uplink with collaborative distribution caching *(SBS-CoDc)* [38]. As its name implies in a no-caching scenario, we have considered the absence of caching at the SBS, while in the cache-assisted uplink, we consider uplink with the support of cache. The simulation parameters are shown in Table 3.

**Table 3:** Simulation parameters.

Parameter name	Value
**MBS**	
Coverage area	1*km*^2^
Frequency band	3.3–4.2 GHz
Location	Outdoor(Top of buildings)
Cache capacity of MBS	128 GB in the range 100–512 GB
Number of contents	500 to 1000
**SBS**	
Number of SBS	20
Location	Indoor/ Outdoor
Coverage area	50 meters
Frequency band	3.6 to 3.8 GHz
Max. distance from MBS	100 to 500 meters
Cache capacity of an SBS cache	32 GB in range 32—128 GB
Number of cache contents	In the range of 200 to 500
**Other parameters**	
Number of MSs	50, 100, 150, 200, 250, 300
Content popularity Levels	10
Transmit direction	Uplink
MS Antenna gain	2 dBi
Cache Replacement Policy	LRU

### 5.1 Performance evaluation metrics

In our system model, *$EC_{U}^{avg}$, $EC_{B}^{avg}$*, and *EC*_*N*_ denote the average energy consumption of the MSs, and SBSs, and the overall network, respectively. In addition, *${ℜ}_{U_{j,i}}^{UL}$, ${ℜ}_{B_{j}}^{UL}$, and ${ℜ}_{Net}^{UL}$* denote the uplink data rate of an MS, an SBS, and the overall network, respectively. For a comprehensive evaluation of the performance of the proposed framework, it is compared with the existing schemes in [33, 38]. The following metrics are used, which are computed as (43)–(55).

#### 5.1.1 Measurements cache availability and efficiency

According to (3) and (4), the cache hit ratio of the overall network is given as

$$\begin{array}{c} {Net}_{{∁}_{hit_{r}}}=\frac{\sum_{j=1}^{M}Hit_{n_{B_{j}}}}{\sum_{j=1}^{M}Hit_{n_{B_{j}}}+\sum_{j=1}^{M}Miss_{n_{B_{j}}}}=\sum_{j=1}^{M}Hit_{r}^{C_{B_{j}}}, \end{array}$$ (43)

where $Hit_{n_{B_{j}}}$ is a cache hit ratio of an SBS *B*_*j*_ and $Miss_{n_{B_{j}}}$ is the cache miss ratio of the same SBS.

While the cache miss ratio of the overall network is given as

$$\begin{array}{c} {Net}_{{∁}_{Miss_{r}}}=\left(1-{Net}_{{∁}_{hit}}\right). \end{array}$$ (44)

#### 5.1.2 Throughput

Throughput (**TH**) is the ratio of the average number of successfully transmitted data in GB per second [70] and is computed as follows:

The throughput of MS (**TH**_*i*_) is given as,

$$\begin{array}{c} {\text{TH}}_{i}=\frac{T_{Tp}^{Num}}{TT}, \end{array}$$ (45)

where $T_{Tp}^{Num}$ is the total number of successfully transmitted data and *TT* is the transmit time.

The average throughput of MSs (${\text{TH}}_{U}^{avg}$) is given as,

$$\begin{array}{c} {\text{TH}}_{U}^{avg}=\frac{\sum_{i=1}^{N}{\text{TH}}_{i}}{N} \end{array}$$ (46)

The average throughput SBSs (${\text{TH}}_{B}^{avg}$) is given as,

$$\begin{array}{c} {\text{TH}}_{B}^{avg}=\frac{\sum_{j=1}^{M}{\text{TH}}_{j}}{M} \end{array}$$ (47)

#### 5.1.3 Energy efficiency measurements

The Energy Efficiency (EE) of the uplink is the ratio of uplink data rate to the energy consumption, where the unit is GB/J/s. Area Energy Efficiency (AEE) is measured based on both the energy consumption and the size of the coverage area, where the unit is GB/Joule per area (*Km*^2^) [66, 67, 71].

In this regard, the average EE of the MSs ($EE_{U}$) can be calculated as,

$$\begin{array}{c} EE_{U}=\frac{\sum_{i=1}^{N}\left({ℜ}_{U_{j,i}}^{UL}/EC_{U_{j,i}}\right)}{N} \end{array}$$ (48)

The average EE of SBSs ($EE_{B}$) can be calculated as,

$$\begin{array}{c} EE_{B}=\frac{\sum_{j=i}^{M}\left({ℜ}_{B_{j}}^{UL}/EC_{B_{j}}\right)}{M} \end{array}$$ (49)

Furthermore, the network AEE is computed in two ways, Firstly, based on the uplink data rate and energy consumption, which is denoted by $\left(AEE_{N}^{ℜ}\right)$ and can be calculated as,

$$\begin{array}{c} AEE_{N}^{ℜ}={ℜ}_{Net}^{UL}/EC_{N} \end{array}$$ (50)

Secondly, based on average EE of MSs and SBSs in addition to the coverage size, which is denoted by $\left(AEE_{N}^{Size}\right)$ and can be calculated as,

$$\begin{array}{c} AEE_{N}^{Size}=\frac{\sum_{j=1}^{M}\sum_{i=1}^{n}EE_{U}+\sum_{j=1}^{M}EE_{B}}{\sum_{j=1}^{M}S_{B_{j}}} \end{array}$$ (51)

#### 5.1.4 Spectral efficiency measurements

Spectral Efficiency (SE) is defined as the uplink data rate per bandwidth measured in GB/s/Hz, which is an important metric to represent the performance of radio resource utilization of the network as given by [67, 72].

According to (19), the average SE for MSs ($SE_{U}^{avg}$) can be calculated as following

$$\begin{array}{c} SE_{U}^{avg}=\frac{\left(\sum_{i=1}^{N}{\text{log}}_{2}\left(1+\frac{TP_{U_{j,i}}^{ul}H_{U_{j,i}}^{ul}{\left\|d\left(U_{j,i},B_{j}\right)\right\|}^{-\alpha }}{\sum_{i\in I_{i}}+\sigma^{2}}\right)\right)}{N} \end{array}$$ (52)

Similarly, the average SE of SBSs ($SE_{B}^{avg}$) can be calculated as following

$$\begin{array}{c} SE_{B}^{avg}=\frac{\left(\sum_{j=1}^{M}{\text{log}}_{2}\left(1+\frac{TP_{B_{j}}^{ul}H_{B_{j}}^{ul}{\left\|d_{B_{j},G}\right\|}^{-\alpha }}{\sigma^{2}}\right)\right)}{M} \end{array}$$ (53)

In addition to, Area Spectral Efficiency (ASE) (*ASE*_*SE*_) (GB/Hz per area *Km*^2^) can be calculated as following

$$\begin{array}{c} ASE_{SE}=\sum_{j=1}^{M}SE_{B_{j}}+\sum_{i=1}^{N}SE_{U_{j,i}} \end{array}$$ (54)

#### 5.1.5 Overall Cache Efficiency (OCE) measurements

The OCE is the ratio of the cumulative cache hits to cumulative demands. That reflects the overall number of cache hits until a specific time (time slot t). An overall cache Efficiency (OCE) is denoted by CO, and is given according to [73] as,

$$\begin{array}{c} OCE=\frac{\sum_{j=1}^{M}{Hit}_{r}^{C_{B_{j}}}}{S_{D}} \end{array}$$ (55)

where, $\sum_{j=1}^{M}{Hit}_{r}^{C_{B_{j}}}$ is the cumulative distributed cache hit ratio, while *S*_*D*_ is cumulative demands.

## 6 Numerical results

The simulation is performed considering the proposed framework and the existing models namely, *No-Cache*, *Each-cache* [33] and *SBS-CoDc* [38]. In addition, their sub-models in different scenarios with the same simulation parameters are listed in Table 3. The final result is validated by the different performance metrics, which are presented in (5.1) and shown below in different Figures.

### 6.1 Cache hit and miss ratio

**Fig 5:**
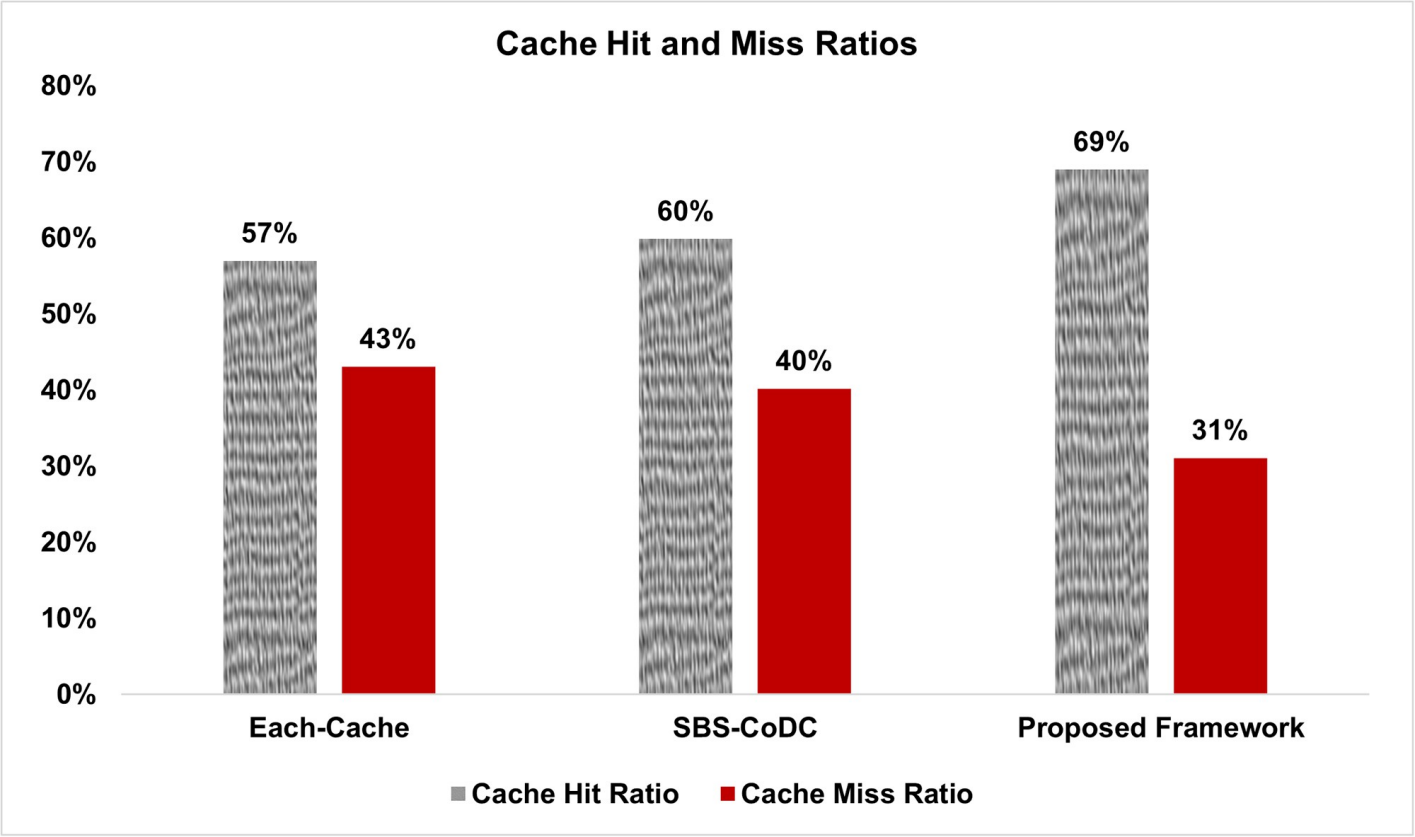
Comparison average cache hit and miss ratios.

Fig 5, shows the average cache hit and miss ratio of the Each-cache, and SBS-CoDc along with our Proposed Framework according to (43) and (44).

We can see a slight increase in the average cache hit ratio of SBS-CoDc as compared to Each-cache because of the lack of cooperation among SBSs. The cache hit ratio of Each-Cache is computed separately and summed subsequently. Our proposed framework outperforms SBS-CoDc by improving the cache hit ratio by 9%. The main reason is the effective use of the CSDP scheme, which improves the cache hit ratio. We can also see that our proposed framework has significantly reduced the cache miss ratio.

The rationale is the distribution of contents among different SBSs along with the UCL, which acts as a map for MSs to efficiently locate the cached contents. Due to these improvements, the traffic load at the access network, as well as backhaul link, is significantly reduced, which subsequently improves EE and SE.

### 6.2 Average Energy Consumption (EC)

**Fig 6:**
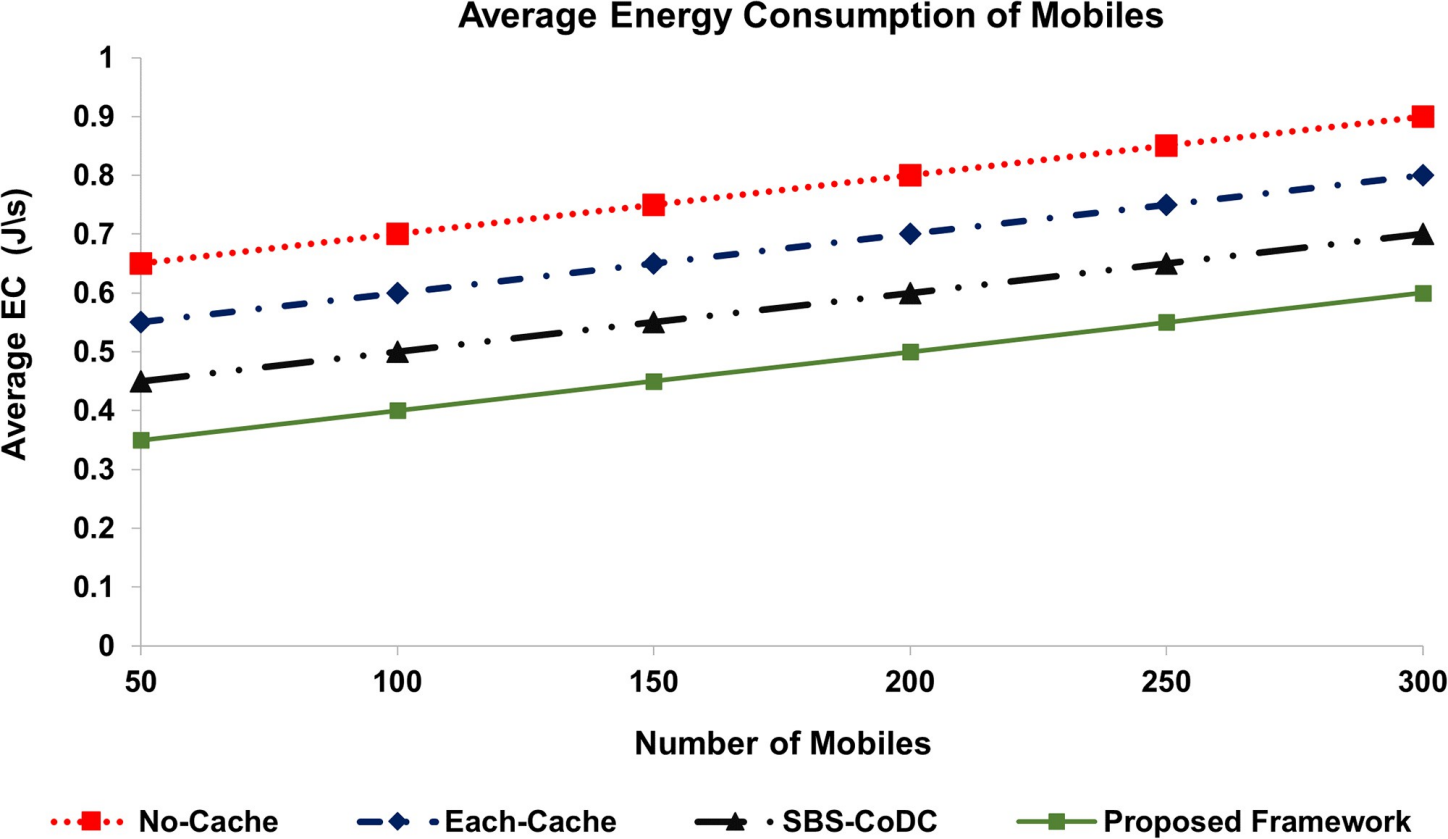
Comparison average energy consumption of MSs.

**Fig 7:**
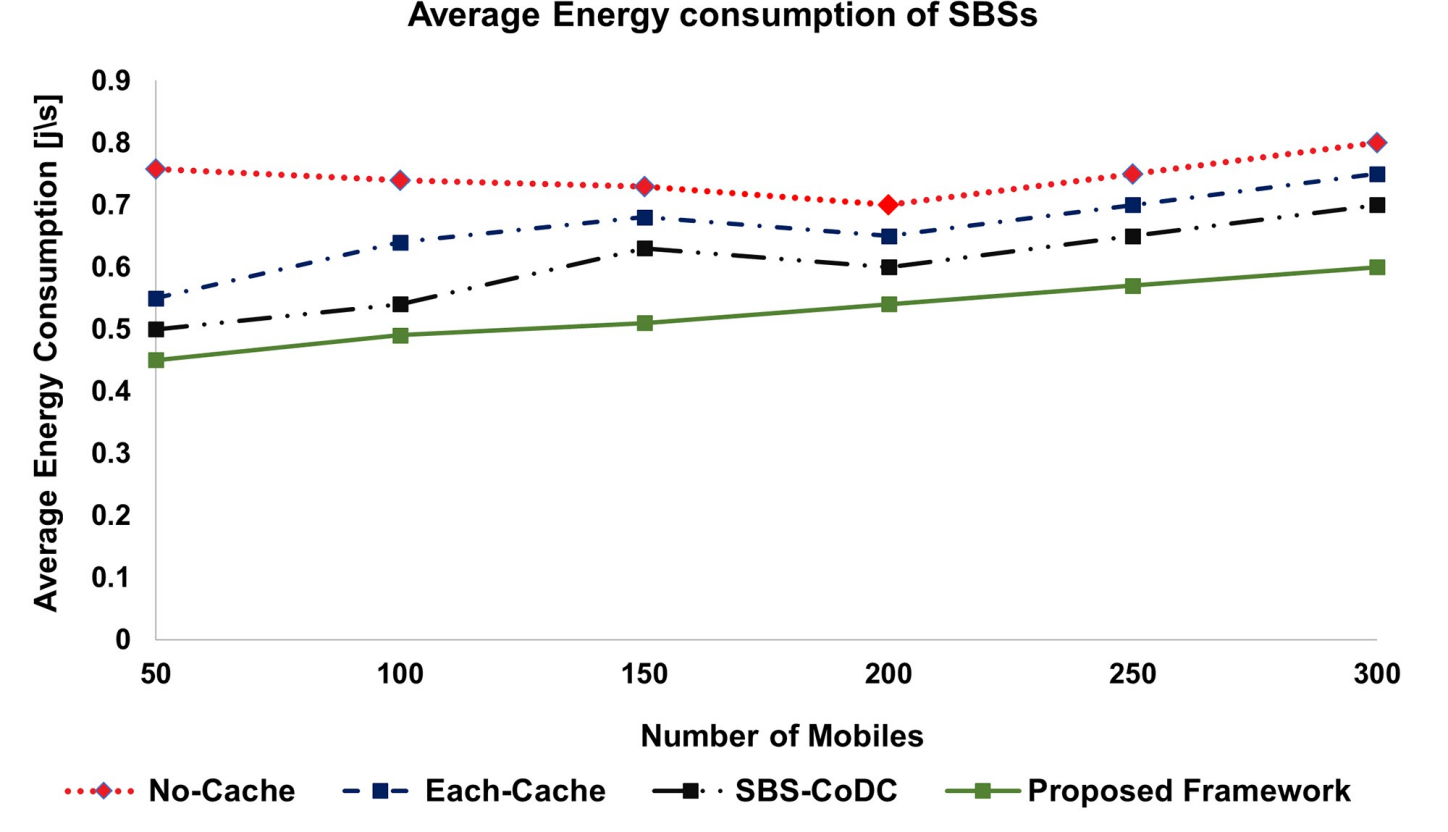
Comparison average energy consumption of SBSs.

The average energy consumption of MSs and SBSs is shown in Figs 6 and 7, respectively. Our proposed framework is compared with No-Cache, Each-cache, and SBS-CoDc. The percentage improvements of average energy consumption of MSs and SBSs are shown in Table 4.

**Table 4:** Percentage improvement of average EC of MSs and SBSs.

Existing Scheme	MS	SBSs	Based on
No-Cache	38.71%	29.46%	(9), (10), (14), (15)
Each-Cache	29.63%	20.4%	-do-
SBS-CoDc	17.39%	12.71%	-do-

We can see that our proposed framework performs significantly better as compared to No-Cache and Each-Cache scenarios. However, about 17% and 13% improvements are noticed as compared to SBS-CoDc, which also has inherent advantages of distributed scenarios. However, our proposed scheme uses UCL for matching at an MS. As the cache hit ratio of our proposed scheme is improved, it also positively affects the average energy consumption by limiting the amount of unnecessary data upload.

### 6.3 Improvement of uplink throughput

**Fig 8:**
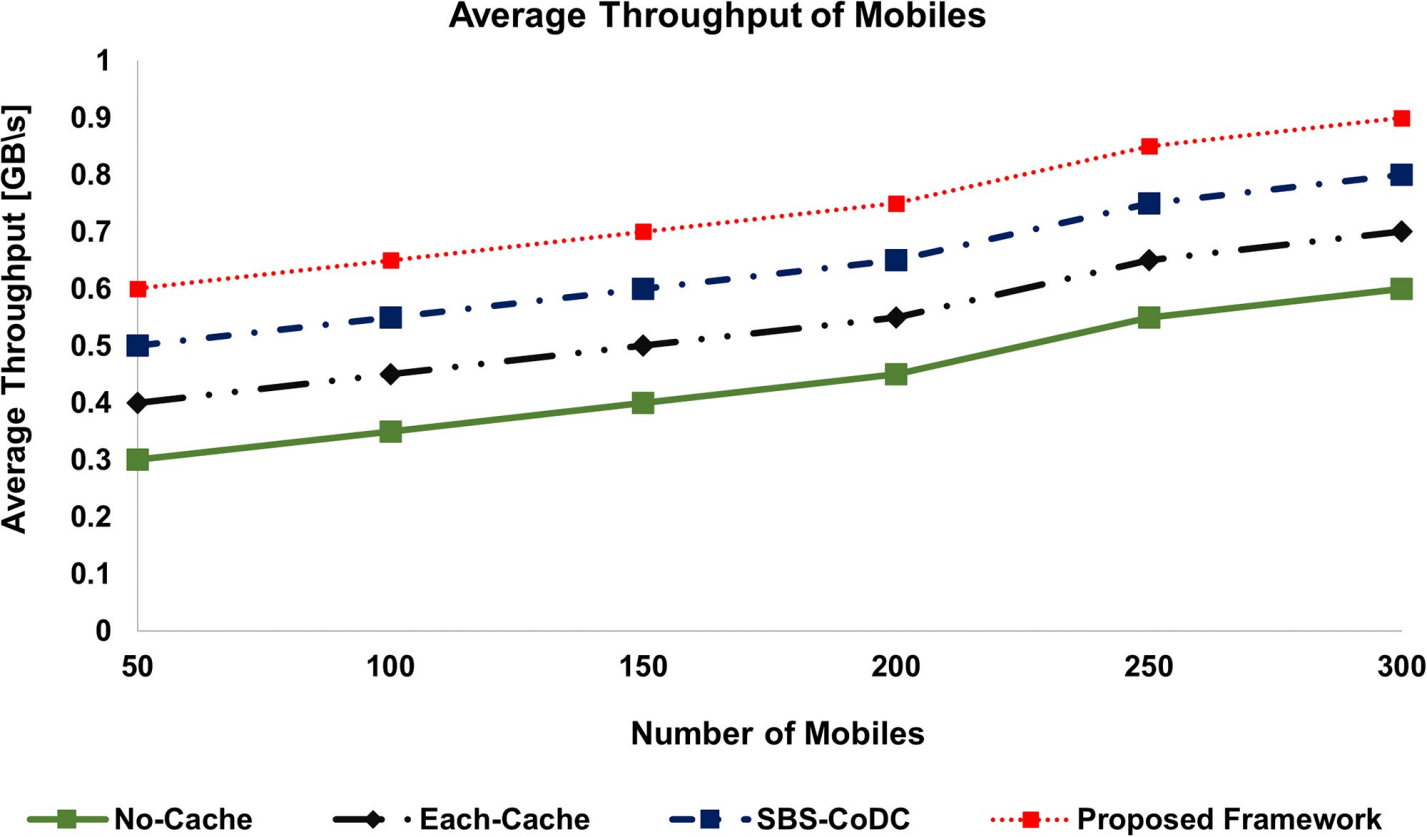
Comparison average throughput of MSs.

**Fig 9:**
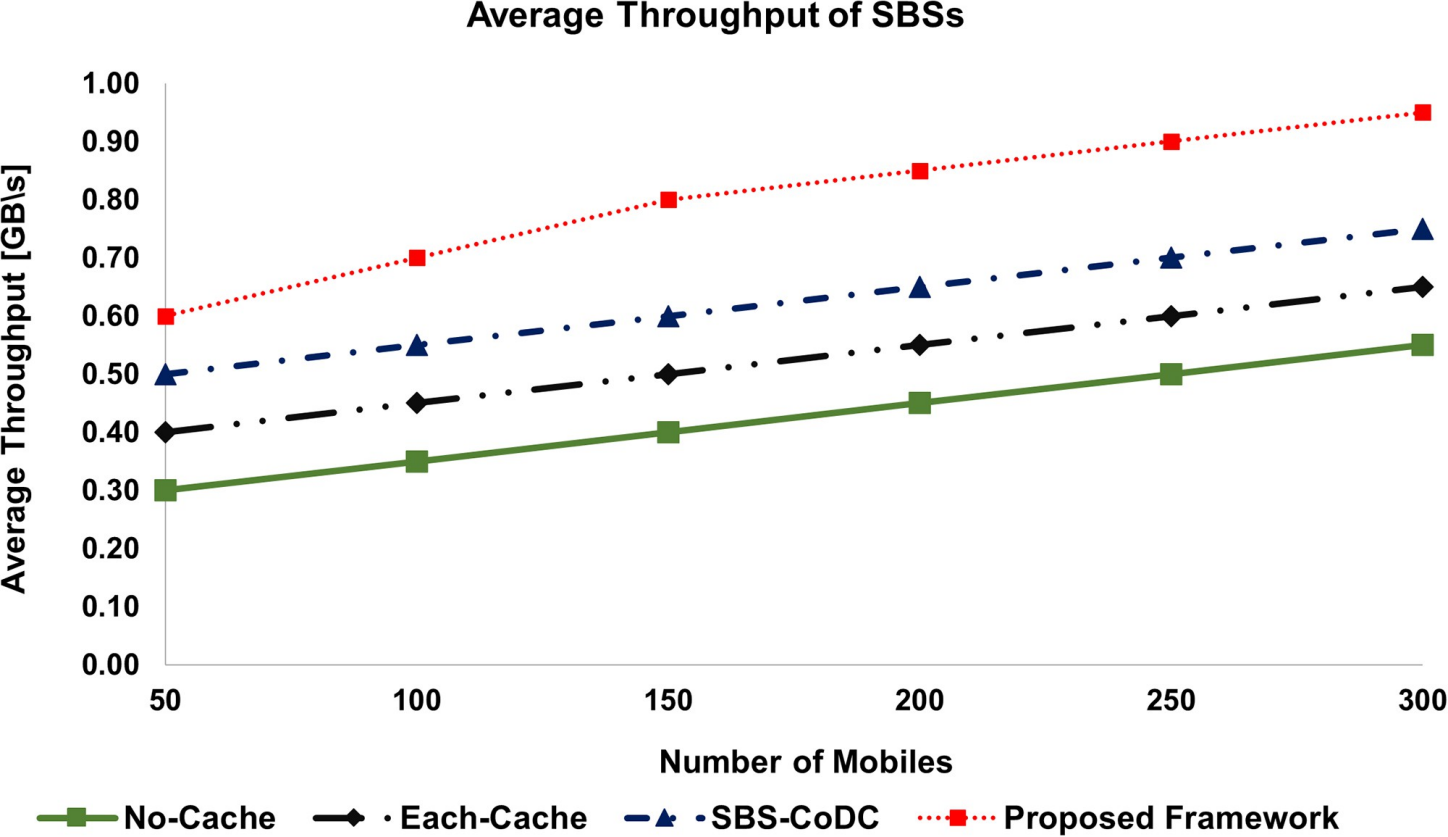
Comparison average throughput of SBSs.

The average Uplink Throughput (**TH**) of our proposed framework is compared with the existing schemes as shown in Figs 8 and 9 of the MSs and SBSs, respectively. The percentage improvements are shown in Table 5.

**Table 5:** Percentage improvement of average throughput of MSs and SBSs.

Existing Scheme	MS	SBSs	Based on
No-Cache	90.04%	88.24%	(46) and (47)
Each-Cache	48.88%	52.38%	-do-
SBS-CoDc	40.00%	28.00%	-do-

We can see that the **TH**s of our proposed framework for both MS and SBS are significantly better than the No-Cache scheme for obvious reasons. Similarly, compared to Each-Cache, our proposed framework performs better due to its distributed nature. The result shows that our framework is better than SBS-CoDc because of the effective use of MoTD rather than uploading new content. Furthermore, as compared to the existing schemes, matching is done at an MS rather than at an SBS, which means no content upload that subsequently improves **TH**.

### 6.4 Improvement in energy efficiency

**Fig 10:**
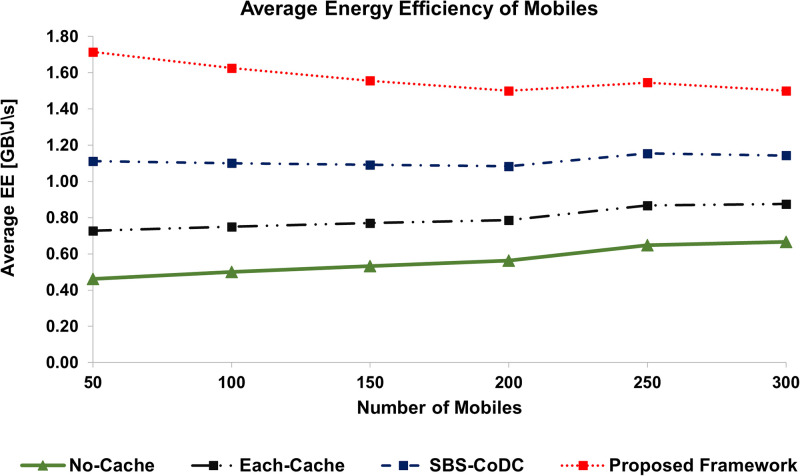
Comparison average EE of MSs among existing schemes.

**Fig 11:**
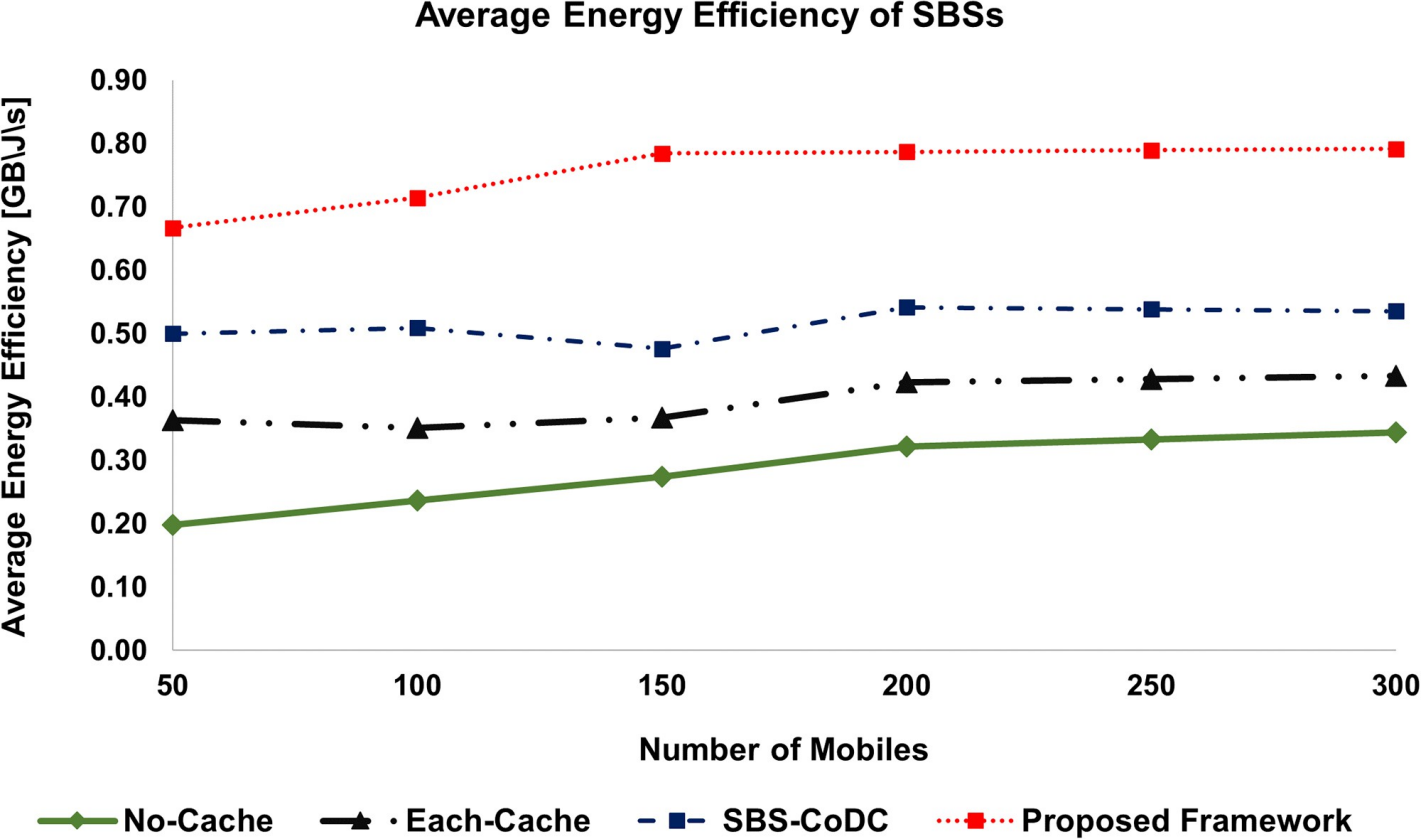
Comparison average EE of SBSs among existing schemes.

Energy efficiency (EE) is an effective way to show performance improvement. As one can predict that the lower energy consumption as shown previously, will also entail the EE. Our proposed framework is evaluated based on EE and compared with the existing schemes. The EE of MS and SBS increased with the increasing number of MSs is shown in Figs 10 and 11. All the percentage improvements of the proposed framework as a result of comparison with existing schemes are shown in Table 6.

**Table 6:** Percentage improvement of average EE of MSs and SBSs.

Existing Scheme	MS	SBSs	Based on
No-Cache	180.04%	165.68%	(48) and (49)
Each-Cache	97.75%	91.46%	-do-
SBS-CoDc	41.28%	46.18%	-do-

We can see in Fig 10, that the average EE of our proposed framework is better as compared to existing schemes. Compared to SBS-CoDc, our proposed framework improves the average EE by 41%. The rationale is the use of UCL for matching at the MS, which effectively reduces the contents upload in case of a cache hit ratio. Similarly, Fig 11, shows the improved average EE of SBSs of our proposed framework. We can see the average EE of our proposed framework as compared to other schemes with an increasing number of MSs. Our proposed framework outperforms existing schemes by significantly improving average EE by 46% as compared to SBS-CODc. The main reason is the improved hit ratio of our proposed framework, which implicitly improves the average EE because of the use of MoTD that represents the new contents.

**Fig 12:**
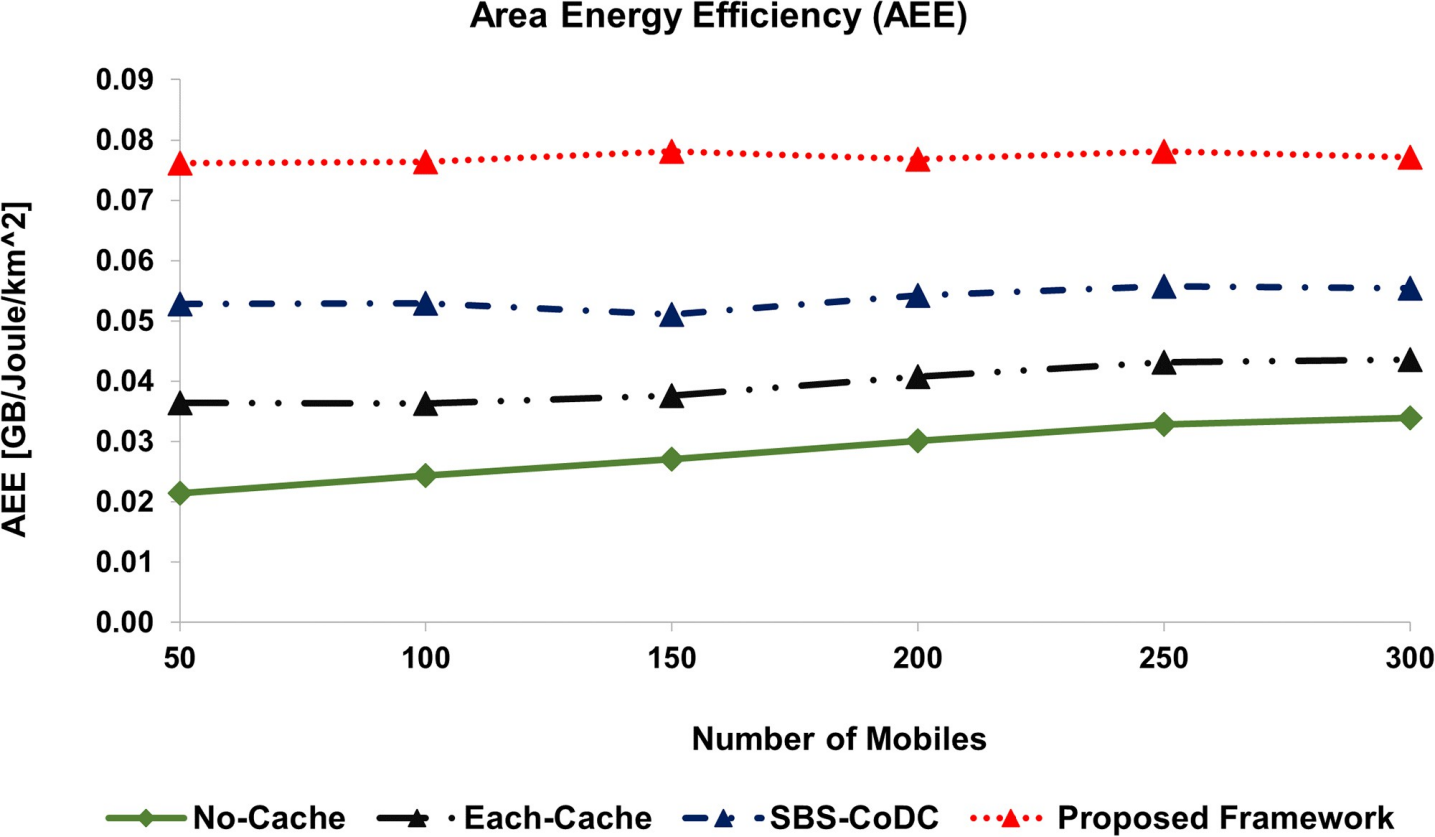
Comparison AEE based on uplink data rate and EC of the overall network.

**Fig 13:**
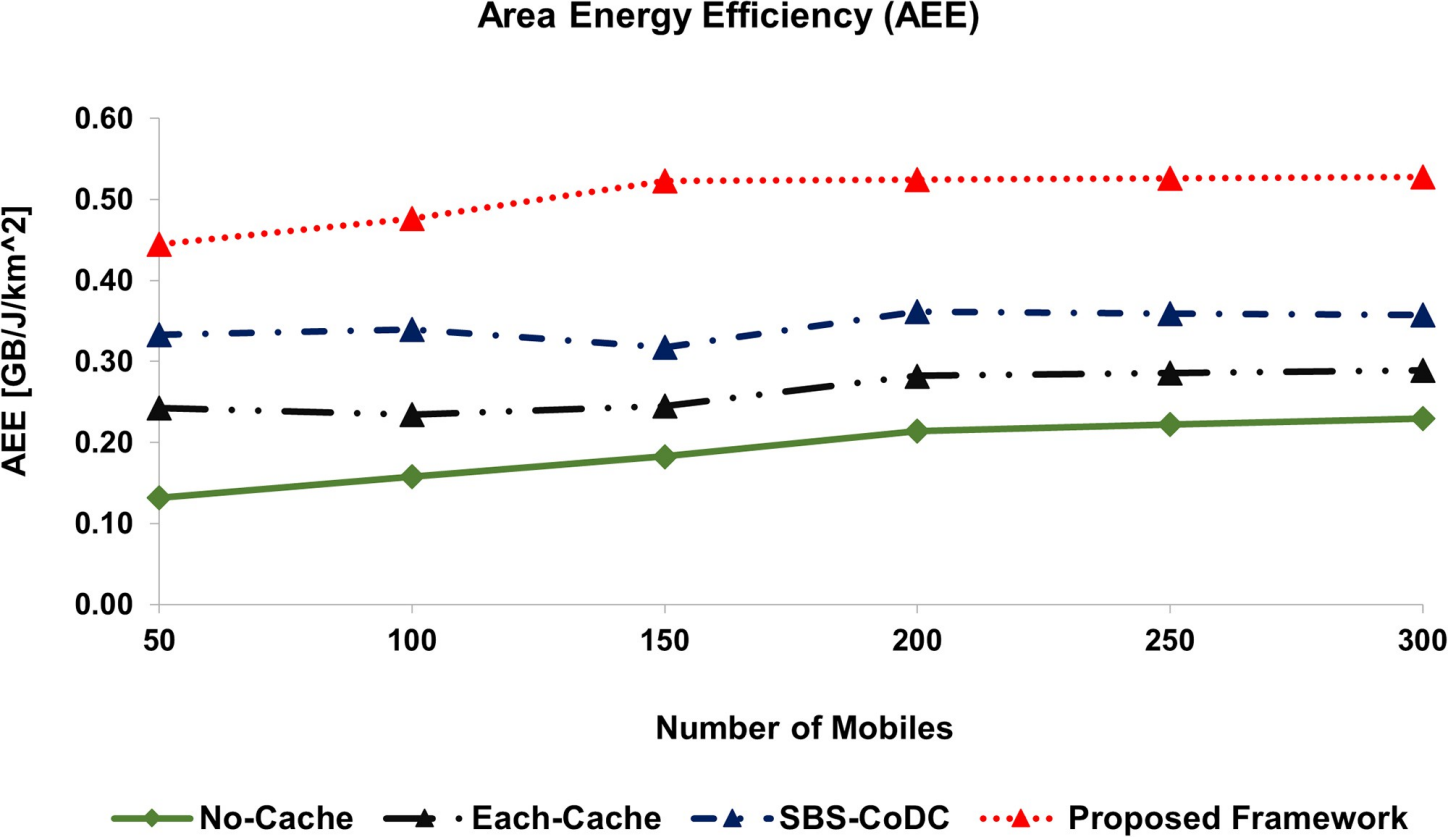
Comparison AEE based on coverage area and EE.

We have also used the metric of Area Energy Efficiency (AEE) to show the performance improvement of our proposed scheme. We computed AEE using two ways, firstly as a function of uplink data and secondly as a function of EE. Figs 12 and 13, show the AEE of No-cache, Each-cache, SBS-CoDc, and proposed framework of different numbers of MSs based on two mentioned ways, respectively.

Fig 12, shows the AEE performance as a function of the uplink data rate, which is computed by dividing the total uplink data rate by the total energy consumption per unit area based on (50). Fig 12, shows that our proposed framework has increased the AEE by 2.34 *GB*/*J*/*km*^2^ as compared to SBS-CoDC. A summary of the result of the comparison with other schemes is shown in Table 7.

**Table 7:** Percentage improvement of average AEE.

Existing Scheme	Uplink Data	EE	Based on
No-Cache	172.44%	172.82%	(50) and (51)
Each-Cache	94.03%	94.62%	-do-
SBS-CoDc	43.35%	43.64%	-do-

Furthermore, Fig 13, shows AEE performance as a function of the cell size, which is computed by dividing the total energy efficiency by the total size of the network based on (51) in order to assess the EE of the overall network to its size. In Fig 13, we can see our proposed framework has increased the average AEE by 43% as compared to SBS-CoDc. These improvements are credited to the improved hit ratio, higher **TH**, and better EE.

### 6.5 Spectral efficiency

**Fig 14:**
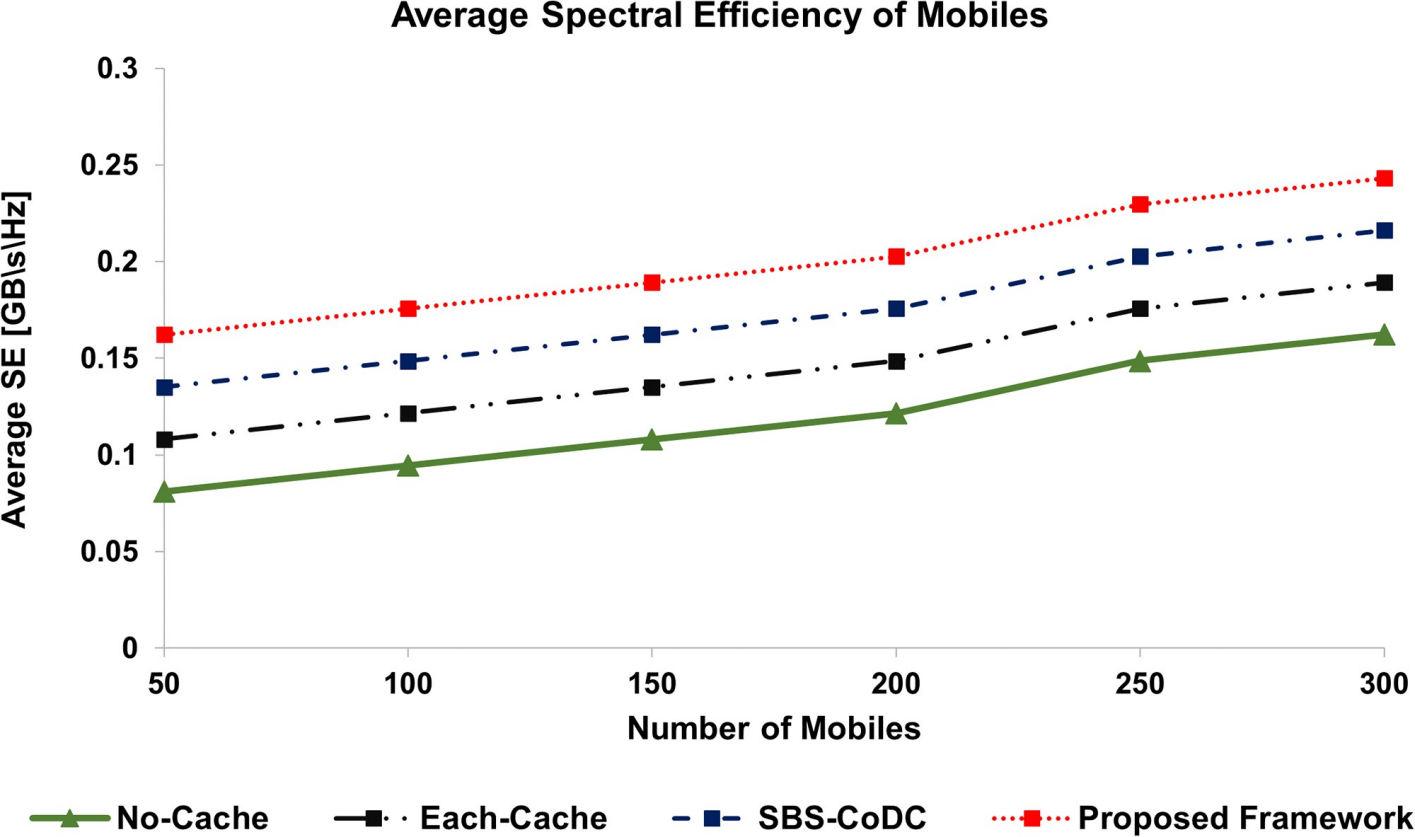
Comparison average spectral efficiency of MSs.

**Fig 15:**
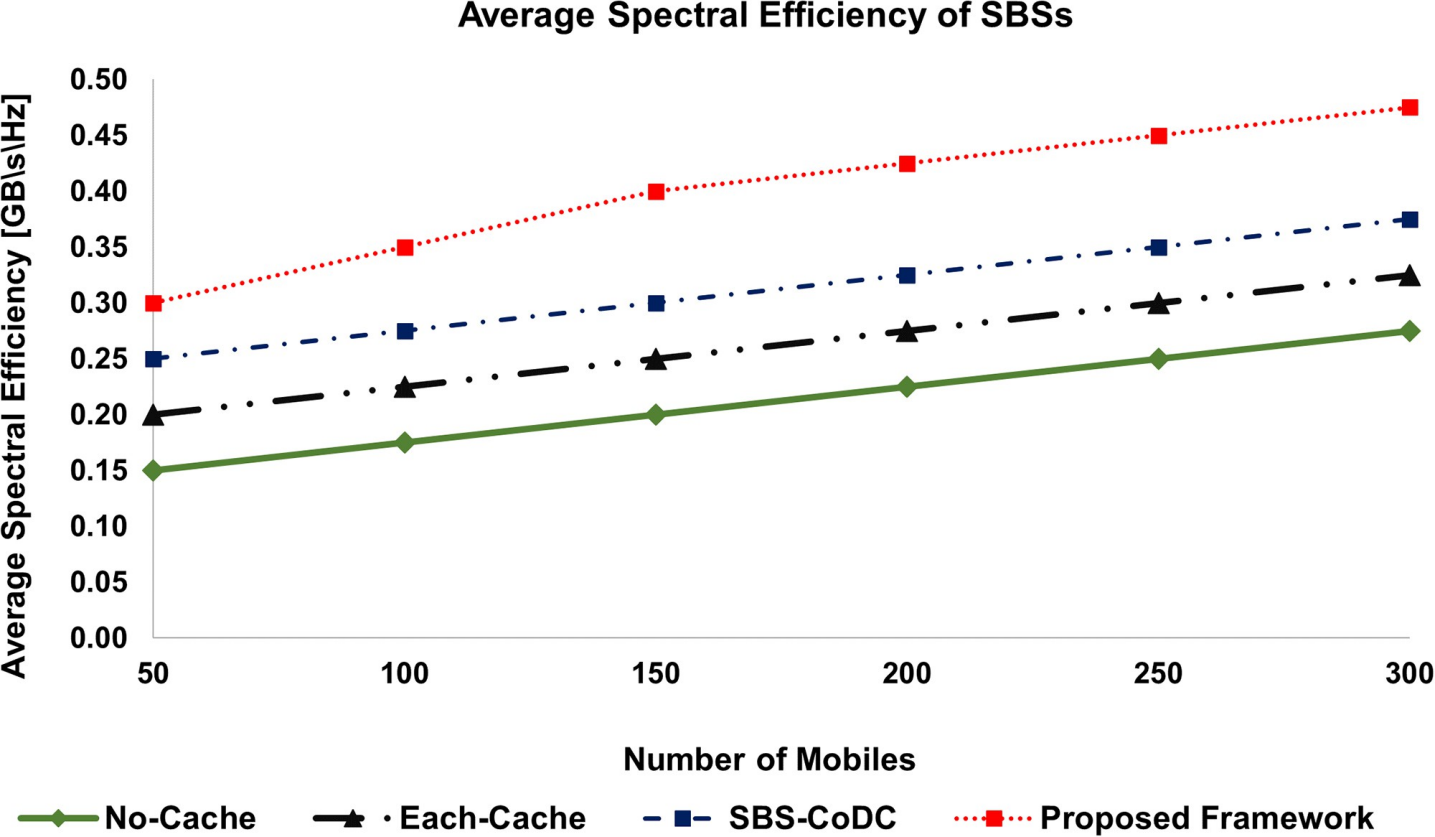
Average spectral efficiency of SBSs as compared to existing schemes.

The average spectral efficiency (SE) of MSs as compared to No-cache, Each-cache, SBS-CoDc, and our proposed framework for different numbers of MSs is shown in Fig 14. We can see that our proposed framework has improved the SE almost by 16% as compared to SBS-CoDc. Whereas, compared to Each-Cache, improvement is almost 37%. Similarly, Fig 15, shows the average SE of SBSs of existing schemes as compared to our proposed framework. The summary of improvement is shown in Table 8.

**Table 8:** Percentage improvement of average SE of MSs and SBSs.

Existing Scheme	MS	SBSs	Based on
No-Cache	67.92%	88.24%	(52) and (53)
Each-Cache	36.92%	52.38%	-do-
SBS-CoDc	15.58%	28.00%	-do-

The rationale of improved SE is a significant reduction in the number of uplink contents. Since matching is done at an MS that facilitates the decision of either uploading the contents or not. In case of a cache hit, the contents are not uploaded and hence the bandwidth is saved for the use of other requests of the remaining MSs. In this way, a significant amount of spectrum can be saved and more requests can be entertained, which ultimately improves the SE.

**Fig 16:**
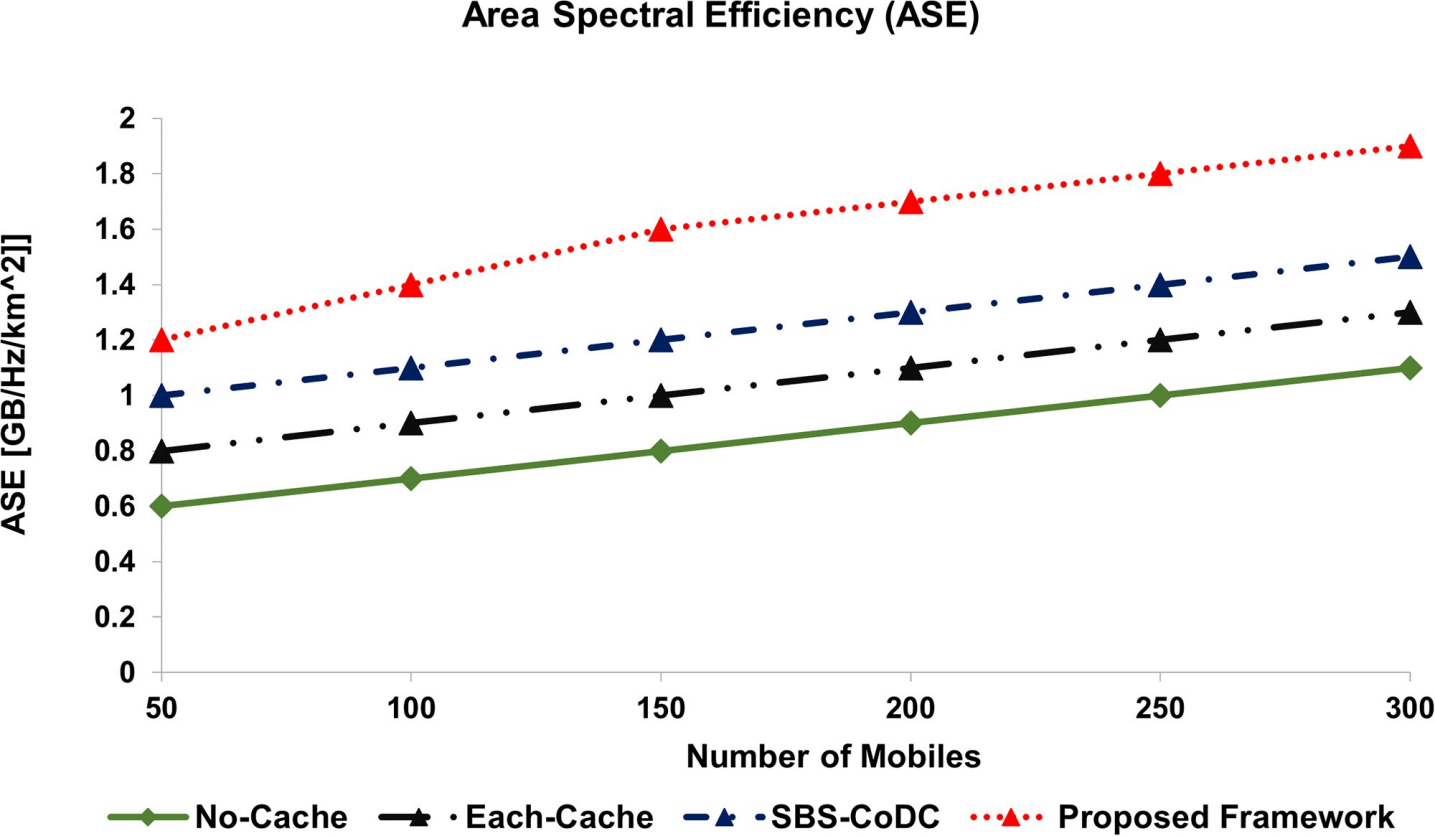
Comparison area spectral efficiency (ASE).

Fig 16, shows the ASE of No-cache, Each-cache, SBS-CoDc, and proposed framework for different numbers of MSs. We can see in Fig 16, that our proposed framework has improved the ASE by 24% as compared to SBS-CoDc.

### 6.6 Improvement of overall distributed cache efficiency

**Fig 17:**
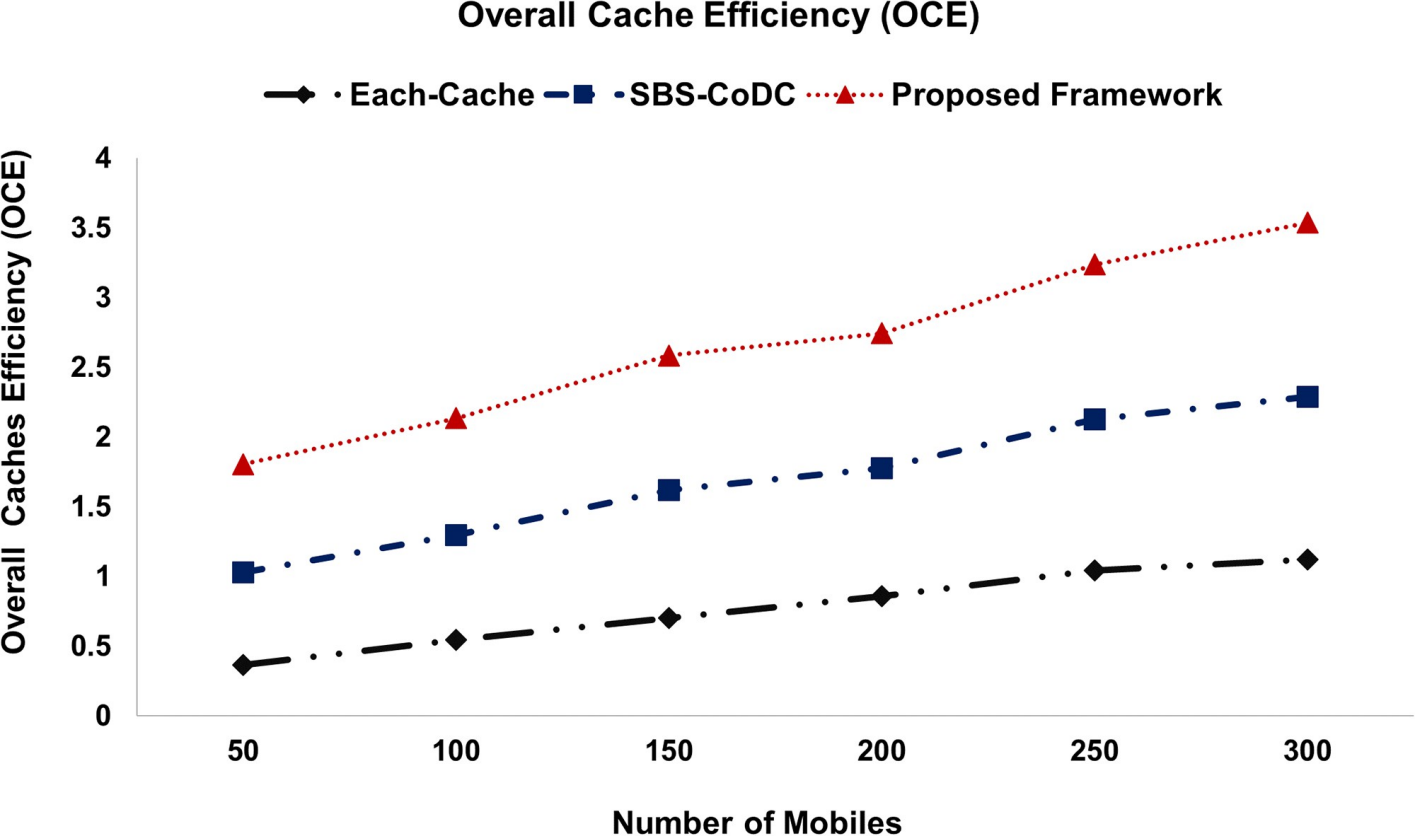
Comparison overall distributed cache efficiency.

The overall cache efficiency (OCE) of the distributed cache as compared to Each-cache, SBS-CoDc, and our proposed framework for different numbers of MSs is shown in Fig 17.

Fig 17, shows the OCE of the distributed cache of existing schemes as compared to our proposed framework. We can see that our proposed framework has improved the OCE almost by 28%, and 7.41% as compared to Each-cache, and SBS-CoDc, respectively. This is because the contents of the distributed cache are available to all the MSs, irrespective of their serving SBSs. In addition, the distributed cache miss ratio is less, which improves the cache efficiency and reduces access time to cache.

## 7 Conclusion

This paper proposed an efficient uplink cache framework based on a distributed scenario. The proposed framework leveraged the content matching at an MS in contrast to the existing schemes, which perform it at an SBS. In addition, the content matching at an MS has significantly improved the energy and spectral efficiency. The rationale is the reduced uplink contents due to local content matching at an MS. Furthermore, the proposed framework is based on the effective distribution of cache contents over cooperative SBSs, which improves the cache hit ratio. This entailed the subsequent improvement in throughput, energy consumption, and spectrum for MSs as well as SBSs. Our analysis shows that our proposed framework improves the EE and SE of the access network by 41.28% and 15.58%, respectively. Furthermore, an increase of 46.18% and 28.00% is respectively calculated as EE and SE for the backhaul link. As well as, the OCE of the proposed framework improves by 28%, and 7.41%, as compared to Each-Cache, and SBS-CoDc, respectively.

## Supporting information

S1 File (ZIP)

S2 File (ZIP)

S3 File (ZIP)

S4 File (ZIP)
